# Enterovirus A71 and coxsackievirus A6 circulation in England, UK, 2006–2017: A mathematical modelling study using cross-sectional seroprevalence data

**DOI:** 10.1371/journal.ppat.1012703

**Published:** 2024-11-20

**Authors:** Everlyn Kamau, Ben Lambert, David J. Allen, Cristina Celma, Stuart Beard, Heli Harvala, Peter Simmonds, Nicholas C. Grassly, Margarita Pons-Salort

**Affiliations:** 1 Nuffield Department of Medicine, University of Oxford, Oxford, United Kingdom; 2 Department of Mathematics, College of Engineering, Mathematics and Physical Sciences, University of Exeter, Exeter, United Kingdom; 3 Department of Comparative Biomedical Sciences, Section Infection and Immunity, School of Veterinary Medicine, Faculty of Health and Medical Sciences, University of Surrey, Guildford, United Kingdom; 4 Enteric Virus Unit, UK Health Security Agency, Colindale, London, United Kingdom; 5 Microbiology Services, NHS Blood Transfusion, London, United Kingdom; 6 Infection and Immunity, University College of London, London, United Kingdom; 7 MRC Center for Global Infectious Disease Analysis, School of Public Health, Imperial College London, London, United Kingdom; National University of Singapore Public Health, SINGAPORE

## Abstract

Enterovirus A71 (EV-A71) and coxsackievirus A6 (CVA6) primarily cause hand, foot and mouth disease and have emerged to cause potential fatal neurological and systemic manifestations. However, limited surveillance data collected through passive surveillance systems hampers characterization of their epidemiological dynamics. We fit a series of catalytic models to age-stratified seroprevalence data for EV-A71 and CVA6 collected in England at three time points (2006, 2011 and 2017) to estimate the force of infection (FOI) over time and assess possible changes in transmission. For both serotypes, model comparison does not support the occurrence of important changes in transmission over the study period, and we find that a declining risk of infection with age and / or seroreversion are needed to explain the seroprevalence data. Furthermore, we provide evidence that the increased number of reports of CVA6 during 2006–2017 is unlikely to be explained by changes in surveillance. Therefore, we hypothesize that the increased number of CVA6 cases observed since 2011 must be explained by increased virus pathogenicity. Further studies of seroprevalence data from other countries would allow to confirm this. Our results underscore the value of seroprevalence data to unravel changes in the circulation dynamics of pathogens with weak surveillance systems and large number of asymptomatic infections.

## Author summary

Enteroviruses commonly cause infections in people of all ages with symptoms ranging from mild to severe. Enterovirus infections create a significant healthcare burden during outbreaks and large epidemics. However, the absence of consistent and systematic surveillance greatly impedes documentation of their true prevalence and collective impact on human health. In the UK, reporting from clinical settings is voluntary and passive, which limits accurate estimation of the overall infection incidence in the population. Here, we use data from seroprevalence surveys, which can detect past infections, to estimate the force of infection (rate at which susceptible individuals acquire infection) of enterovirus A71 and coxsackievirus A6 in England, UK, between 2006 and 2017. We found that transmission intensity for both enterovirus serotypes has remained relatively unchanged in this 12-year period. Furthermore, we analyze changes in serotype-specific enterovirus detections in England over the same period and found evidence that the increased number of reported CVA6 detections cannot only be related to changes in testing intensity. We therefore conclude that the increase in CVA6-related disease cases across England during this period could be attributed to the acquisition of increased pathogenicity (probability of disease after infection) of this serotype. Our approach may be useful to study the population-wide infection burden of other enterovirus serotypes for which clinical surveillance is limited.

## Introduction

Human enterovirus A71 (EV-A71) and coxsackievirus A6 (CVA6) are widespread pathogens mainly transmitted through the fecal-oral route but also through respiratory droplets and indirect contact with surfaces and objects contaminated by excretions from infected persons [1]. EV-A71 and CVA6 (jointly with Coxsackievirus A16) are members of the species Enterovirus A in the family *Picornaviridae* [2] and are the main causes of hand, foot and mouth disease (HFMD), a disease associated with rash on hands and feet and vesicles in the mouth, usually accompanied with fever. HFMD affects mostly children under the age of 5 years old and is typically mild and self-limiting but is occasionally linked to potentially fatal neurologic and severe systemic manifestations [1]. Outbreaks of HFMD typically occur in nurseries and schools, and nursery and school closure is used to halt transmission in some countries [3]. This consequently leads to disruptions to parents and caregivers, potentially imposing an important economic burden [4].

HFMD outbreaks tend to occur every summer in temperate regions [5,6] but have a less clear seasonality in the tropics, as seen in Singapore [7]. Both EV-A71 and CVA6 exhibit cyclic circulation patterns, which have been shown to be driven by the acquisition of serotype-specific immunity [8]. For example, EV-A71 circulation peaked every 2–3 years in Malaysia [9] and Japan [8], and CVA6 circulation peaked every 2 years in Japan [8].

EV-A71 has been the primary focus of HFMD surveillance and vaccine development [10] as it is responsible for most severe cases of HFMD. 93% of deaths were associated with this serotype in an observational study from China published in 2014 [6]. Although the first epidemics of EV-A71 were reported in the early 1970’s, the first large EV-A71-associated HFMD epidemics were not recorded until 1997 in Malaysia (with 2628 HFMD cases, including 29 deaths due to encephalitis and cardiac failure) [11] and 1998 in Taiwan (with an estimated 1.5 million people infected and children admitted to hospital for serious neurological complications) [12]. Sporadic EV-A71 outbreaks or epidemics associated with neurological complications mostly in children have also been reported, including in Spain in 2016 [13], in Germany in 2019 [14] and in Colorado, USA, in 2018 [15].

CVA6 emerged worldwide in the late 2000’s and early 2010’s surpassing EV-A71 and CVA16 as the leading cause of HFMD, and is a growing public health concern [16–20]. In 2008 to 2010, CVA6 was associated with widespread HFMD infections in Finland, Italy, China, US, UK and Spain [21–25], characterized by high fever, generalized vesiculobullous lesions that ulcerate and scab, and onychomadesis (nail shedding) mostly in young children, in addition to occasional cases of viral meningitis and encephalitis [26]. CVA6 infections are now frequent in most countries, and in Europe, CVA6 was the most frequently reported enterovirus type between 2015–2017, representing 13% of typed enteroviruses [27].

**Fig 1:**
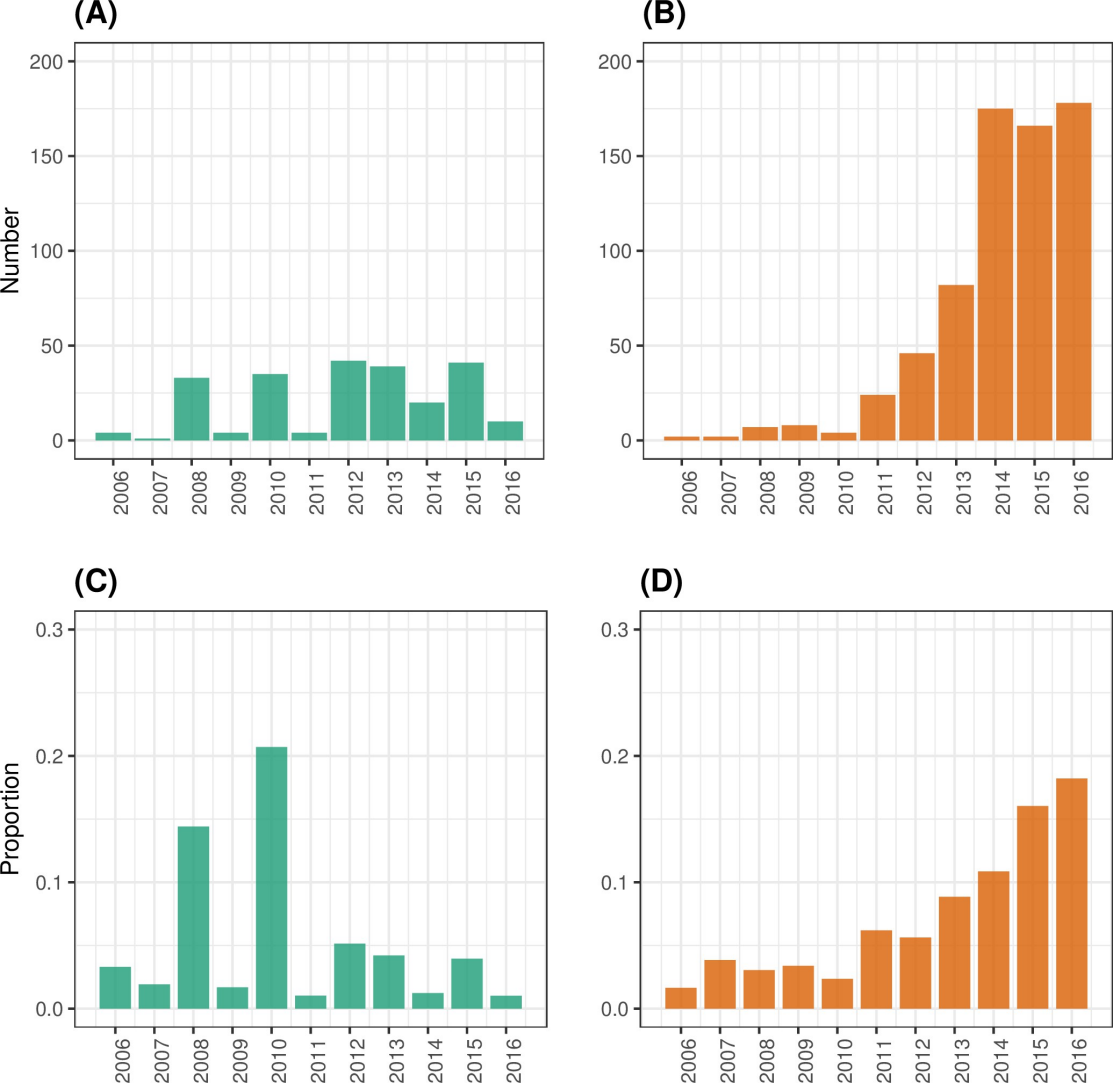
EV-A71 and CVA6 detections among all genotyped enterovirus-positive referrals in England, UK, between 2006 and 2017. The number of EV-A71 (A) and CVA6 (B) detections among all genotyped enterovirus-positive referrals submitted for genotyping to UKHSA are shown, as well as their respective contribution to the overall genotyped enterovirus-positive referrals each year in (C) and (D), respectively.

In England, between 2006 and 2016, the number of laboratory-confirmed EV-A71 detections reported by health authorities shows a biennial pattern, as well as a slight general increasing trend between 2006 and 2013 (Fig 1A), with the highest years reporting no more than 50 detections. The number of laboratory-confirmed CVA6 detections, however, shows a clear increasing trend over time (Fig 1B), coinciding with the emergence of this pathogen globally, with total numbers surpassing 150 detections per year since 2014 in England. The general increase in reported cases of both serotypes, EV-A71 and CVA6, may be explained partly by improved enterovirus surveillance. However, it is unclear whether transmission has also increased during this period.

Characterizing virus transmission dynamics is critical for assessing their causal relationship with disease syndromes, quantifying their severity, and potentially predicting the course of outbreaks. This is dependent on consistent surveillance systems with consistent and thorough temporal and spatial reporting of virus detections. However, as enterovirus infections are commonly subclinical and case notification is based on passive surveillance systems in most countries [28,29], these data are sparse and provide insufficient information to characterize enterovirus dynamics. Seroprevalence studies can identify symptomatic and asymptomatic past infections and so provide an alternative and cost-effective way to reconstruct historical patterns of infection and for identifying undetected outbreaks or sustained transmission [30].

Here, we combine virus-specific age-stratified seroprevalence data from a seroepidemiology study conducted in England [31] and mathematical models to assess changes in transmission of EV-A71 and CVA6 between 2006 and 2017 in this country.

## Material and methods

### Virological data

The UK Health Security Agency (UKHSA, formerly Public Health England (PHE)) has an established national Enterovirus Surveillance System (ESS) hosted at the Virus Reference Department, UKHSA, Colindale. Here, specimens (including stool, swabs, cerebrospinal fluid (CSF) and respiratory samples) in which an enterovirus has been detected are sent for enterovirus genotyping. These specimens are primarily from but not limited to individuals who presented to hospitals with acute neurological syndromes. The referral of these enterovirus-positive infections to UKHSA is done on a voluntary basis, and symptoms severity likely influences which patients are sampled, and which specimens are tested for enterovirus and ultimately referred to UKHSA for genotyping. We accessed ESS data collected between 2006 and 2017 on enterovirus genotype, sample type, year of sample collection and geographical data on location of the case. Geographical information was aggregated as the city of the laboratory from which the primary specimen was referred, and so these cities may represent a wider regional geographic area beyond the metropolitan area of the city itself.

### Age-stratified seroprevalence data

Deidentified archived serum samples were obtained from the SeroEpidemiology Unit of UKHSA. The samples were collected from the general population, all ages from 0 to 92 years, in England in 2006 (n = 514), 2011 (n = 498) and 2017 (n = 561). Samples were labeled with age and date of collection. The three cross-sectional surveys (in 2006, 2011 and 2017) represent snapshots of seroprevalence at three time points when different levels of virus were detected in the population. The 2006 cross-section represents a period when few EV-A71 and CVA6 clinical infections were reported in England (32 clinical specimens collected between 1998 and 2006 were positive for EV-A71 [32], and only three specimens collected between 2004 and 2006 were CVA6 positive [33]). The 2011 timepoint was at the start of a wave of increases in virus detection from 2011 to 2016 (Fig 1), and the 2017 timepoint was used to measure seroprevalence following this increase. Neutralizing antibody titers were determined by live-virus neutralization assays using RD cells, a standard amount of virus (a B4 genogroup/2002 and a 2016 strain, respectively for EV-A71 and CVA6) and serial 2-fold dilutions of serum as previously described [31]. Serum neutralization titers were determined by the presence of cytopathic effect. Further details of the seroepidemiological study can be found in [31].

### Statistical analyses

The force of infection (FOI) describes the rate at which a susceptible person acquires an infection and is a key parameter in infectious disease epidemiology. Catalytic models use age-stratified seroprevalence data to estimate the FOI [34]. Here, we fit six catalytic models to the three cross-sectional EV-A71 and CVA6 serosurveys to explore whether the seroprevalence data supports changes in transmission over time. Model 1 is the simplest model, which assumes that individuals seroconvert at a constant rate λ and remain seropositive throughout their lives (S1 Text). Under this model, the proportion of individuals who have seroconverted at age *a* is given by [35]:

$$z(a)=1-e^{-\lambda a}$$

Model 2 is the reversible catalytic model, which assumes that individuals who have seroconverted can serorevert (become seronegative) at a constant rate *ρ*, where the duration of antibody detection follows an exponential distribution with a mean of 1/*ρ*. In this model, the proportion of individuals seropositive at age *a*, as described in [35] is:

$$z(a)=\frac{\lambda }{\lambda +\rho }(1-e^{-a(\lambda +\rho )}),$$

with seropositivity plateauing at $z(\infty )=\frac{\lambda }{\lambda +\rho }$. We extend Models 1 and 2 by allowing the FOI to be initially constant over time (*λ_c_*), and then from year *T* (fixed to 2006, the first year with seroprevalence data) to change over time following a random walk (*λ_t_*) with a time step of 1 year (Models 3 and 4):

$$\lambda (t)=\left\{ \begin{array}{c} \lambda_{c},t<T\\ \lambda_{t},t\geq T \end{array}\right.$$

See S1 Table for model parameterization and the S1 Text for the full specification of Models 3 and 4.

These four models assumed the same risk of infection across age. However, the relative risk of infection is likely to change with age for the two serotypes, given that HFMD occurs mainly in children under 5 years. Therefore, we additionally explored whether there was evidence of an FOI which declines with age by extending the time-constant FOI models above, such that: $\lambda (a)=\lambda_{1}e^{-\beta (a-1)}$, where *λ*_1_>0 and *β*>0. Model 5 denotes the time-constant model with an age-dependent FOI and no seroreversion and Model 6 denotes the same model with seroreversion.

We fitted all six models to the data using a binomial likelihood:

p(a)∼Binomial(n(a),z(a))

where *p(a)* is the number of seropositive individuals at age *a*, *n(a)* is the number of individuals of age *a* who were tested, and *z(a)* is the proportion seropositive at age *a* under the Models 1–6. The models were fit to the data for the first 80 age classes (ages 1 to 80 years). We ensured that all observations for a given serotype were independent and did not come from the same individual. To avoid the effect of maternal antibodies, we excluded seroprevalence data for individuals <1 year of age, and individuals were allowed to be infected only from age 1. We also assumed the serology assays uncovered seropositivity with 100% accuracy. Although microneutralisation assays for enteroviruses are considered highly specific and sensitive, we conducted a sensitivity analysis assuming imperfect assay accuracy (S1 Text).

We report the estimated annual probability of infection or attack rate for year *t*, *p*_*t*_, which is the proportion of the susceptible population that becomes seropositive each year and can be derived from the FOI estimate as follows:

$$p_{t}=1-e^{-\lambda_{t}}$$

The models were fit under a Bayesian paradigm, and the priors specified for the parameters are given in S1 Table. Across the model types, we set priors for *λ* which were uninformative with respect to the annual probability of infection. For the time-constant FOI models (Models 1, 2, 5 and 6), we chose a prior on *λ* which corresponded to a uniform prior over [0, 1] for the annual probability of infection. We used the same prior for the initial FOI value for the time-varying FOI models (Models 3 and 4) and a random walk prior on the FOIs from then on. For Models 2, 4 and 6, which included seroreversion, we used an exponential prior for the seroreversion rate (*ρ*) and assumed that the typical duration of immunity was 20 years, based on a modeling study of non-polio enteroviruses that estimated the duration of protective immunity to be long-lasting on the order of multiple years [8]. We also performed sensitivity analyses on the prior of *ρ* to test a shorter duration of seropositivity (S1 Text). S1 and S2 Figs show the prior predictive simulations of the age-profile of seropositivity for Models 1, 2, 5 and 6. We do not show prior predictive simulations for Models 3 and 4 since these models only allow extra variation in *λ* over time and above the constant FOI models. The plots in S1 and S2 Figs illustrate that a wide range of seropositivity profiles were possible given our choice of priors. We also conducted prior sensitivity analyses where we explored how model fitting and parameter estimation varied for differing priors on *λ* (see S1 Text), and this showed that the results were insensitive to the choice of priors on *λ*.

The models were specified and fit using Stan via the *RStan* package, which implements the Monte Carlo No-U-Turn-Sampler algorithm to explore the posterior parameter distributions [36]. We ran four chains in parallel for 10,000 iterations each with a warmup of 3,000 iterations per chain. Convergence was assessed visually using MCMC trace plots, effective sample size (ESS) and Gelman-Rubin’s R-hat diagnostic. A threshold of 1.01 was used to diagnose convergence for the R-hat diagnostic. The posterior distributions were summarized by their means and 2.5^th^ and 97.5^th^ percentiles reported as 95% credible interval (CrI). Model comparison analysis was performed using Pareto-smoothed importance sampling leave-one-out information criterion (PSIS-LOOIC) with the loo R package (https://mc-stan.org/loo/).

## Results

### Assessing changes in genotyped enterovirus-positive referrals submitted to UKHSA

As enterovirus surveillance in the UK is passive, unrecognized changes in testing and referral for characterization to UKHSA (which could occur for many reasons) may potentially explain the temporal trends in the total number of EV-A71 and CVA6 positive detections collected by the ESS (Fig 1A and 1B). To evaluate the evidence for this during the study period, we examined metadata (including serotype detected, city reporting and sample type) of laboratory-confirmed enterovirus-positive infections submitted by National Health Services laboratories in England to UKHSA between 2006 and 2017.

There was a general increase in genotyped enterovirus-positive referrals submitted to UKHSA (all serotypes combined) over time, with a peak in 2014 (S3 Fig), likely due to increased awareness of enterovirus-associated illness and expanded virus surveillance that resulted from the global EV-D68 outbreak that year [37]. Furthermore, the contribution of EV-A71 to the total genotyped enterovirus-positive referrals showed a biennial pattern, probably reflecting cyclical circulation of this serotype, with the maximum contribution years in 2008 and 2010, representing 14% and 21% of the total genotyped enterovirus-positive referrals respectively (Fig 1C). In contrast, the contribution of CVA6 increased over the study period from only 1.6% in 2006 to 17% in 2016 and 2017 (Fig 1D). For comparison, the contribution of CVA16 (which jointly with EV-A71 and CVA6 is one of the main serotypes responsible of HFMD) remained relatively flat, fluctuating between 0.8% and 4.8% annually, except for 2011, where it represented 10% of all genotyped enterovirus-positive referrals (S4 Fig). Despite the global EV-D68 outbreak in 2014, the contribution of EV-D68 to the total number of genotyped enterovirus-positive referrals that year was only 3% (S4 Fig), suggesting that the increase in testing resulted in an increase of detections of other serotypes that circulated in 2014.

To investigate possible geographical changes in reporting, the number of genotyped enterovirus-positive referrals submitted from the cities in England that submitted at least 90 enterovirus-positive detections over the study period were examined (S5 and S6 Figs). In agreement with nationally aggregated data, the number of genotyped enterovirus-positive referrals was highest in 2014 in most cities (S5 Fig). Several cities did not refer enterovirus detections in the first few years (e.g., Birmingham and Brighton) or showed a clear increase in detections over time (e.g., London and Sheffield), both contributing to the general increase at the national level. Bristol, Manchester and London, with a total of 1726, 1489 and 905 genotyped enterovirus-positive referrals reported between 2006 and 2017 respectively, were the three cities that reported most detections.

Changes in sample type reported over time could indicate a change in enterovirus-related disease during this period, or changes in clinical practice or diagnostic procedures. Information on sample type has been available for >75% of all genotyped enterovirus-positive referrals each year (S7 Fig). The four most reported sample types, by decreasing order, were CSF, stool, respiratory sample and skin swab. There was a clear increase in skin swab samples over time (S8 Fig), with an increasing proportion of those being positive for CVA6, and above 85% since 2012 (S9 Fig). This could indicate an increase in HFMD cases associated with CVA6.

These data suggest an expansion of enterovirus surveillance from around 2012, indicated by the larger number of genotyped enterovirus-positive referrals reported annually by the ESS since then, and a general improvement in the quality of surveillance data, as shown by the higher proportion of detections with available information on sample type. Despite the general increase in the number of genotyped enterovirus-positive referrals (all serotypes combined) observed between 2006 and 2017, the consistent increase in the contribution of CVA6 to the total number of enterovirus detections, in addition to the increase in the number of skin swabs positive for CVA6, provide evidence that the number of HFMD cases associated with CVA6 may had substantially increased in the period 2006–2017. In contrast, it was difficult to determine whether there were substantial changes over time in the number of EV-A71 cases. This serotype contributed to a high proportion of the total genotyped enterovirus-positive referrals in 2008 and 2010 when the total number of referrals was small but has since contributed a smaller and relatively unchanging proportion.

### Antibody titers and age-stratified seroprevalence

**Fig 2:**
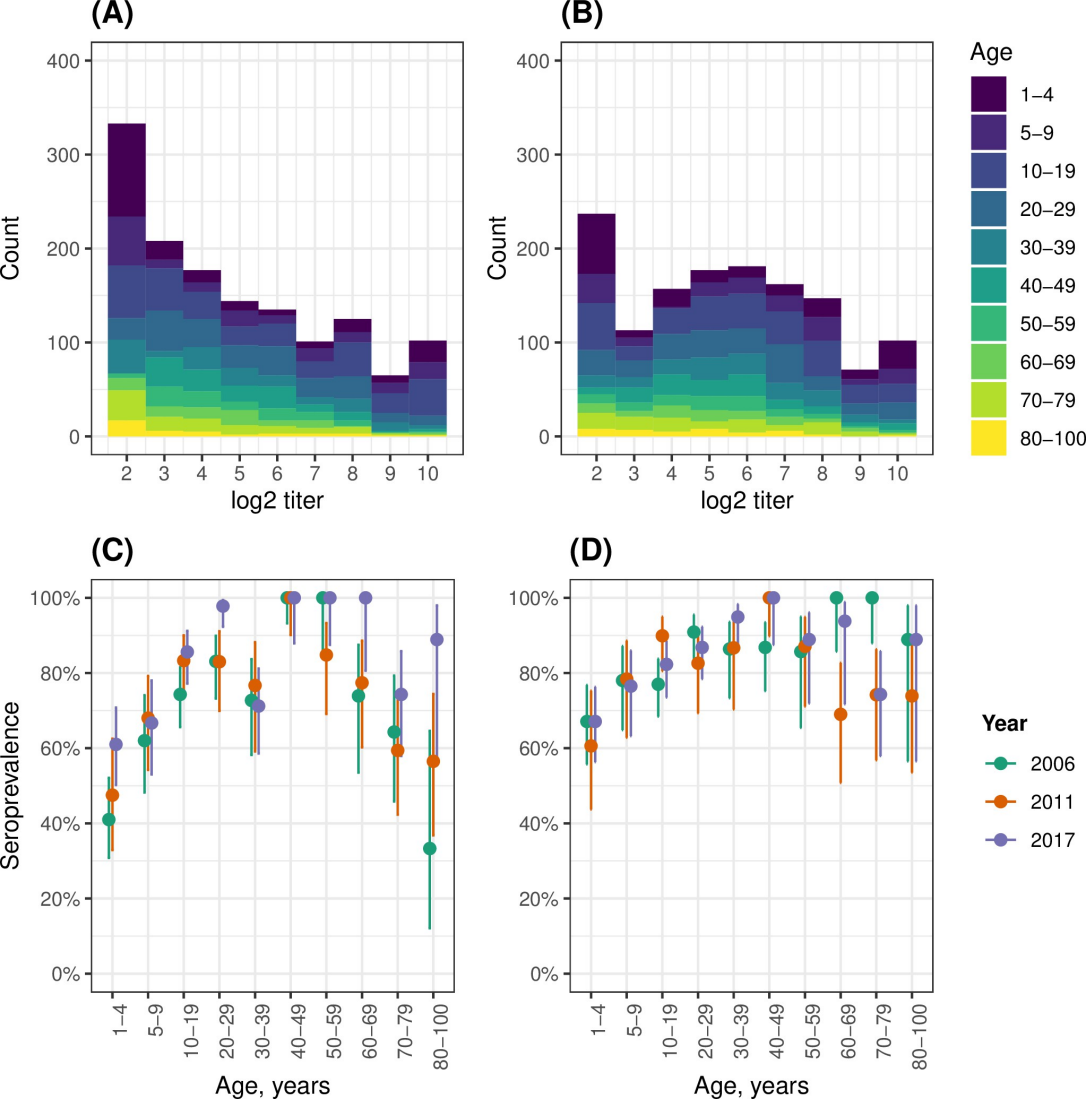
Distribution of antibody titers and age-stratified seroprevalence. Distribution of log2 virus neutralization titers against EV-A71 (A) and CVA6 (B) obtained from three cross-sectional serosurveys (2006, 2011 and 2017) combined. Also shown is EV-A71 and CVA6 seroprevalence by age class for EV-A71 (C) and CVA6 (D) in the three cross-sectional serosurveys in England, UK. For both serotypes, a seropositivity cutoff of ≥1:8 antibody titer was used. Vertical lines represent 95% binomial confidence intervals.

The CVA6 antibody titers show a clear bimodal distribution with a trough at 1:8 (Figs 2B and S10B), suggesting that an antibody titer of ≥1:8 may be a reasonable cut-off to define seropositivity. For EV-A71, however, the antibody titer distribution is not bimodal, but it shows a disproportionately high frequency for the 1:8 endpoint (Figs 2A and S10A). When restricting to the younger population, this disproportion is even clearer (S10C Fig). Therefore, we also use the ≥1:8 seropositivity cut-off for EV-A71, in agreement with other studies [38,39].

Both serotypes showed a rapid increase in seropositivity in younger years, with the highest levels of seroprevalence reached in the 40–49 age class (Fig 2C and 2D, S2 Table). This was followed by a plateau for CVA6 (Fig 2D) and by a decline for EV-A71 (Fig 2C). Notably, there were no major differences in the seroprevalence age-profiles across the samples collected in 2006, 2011 and 2017.

### No evidence for important changes in the FOI over time

**Fig 3:**
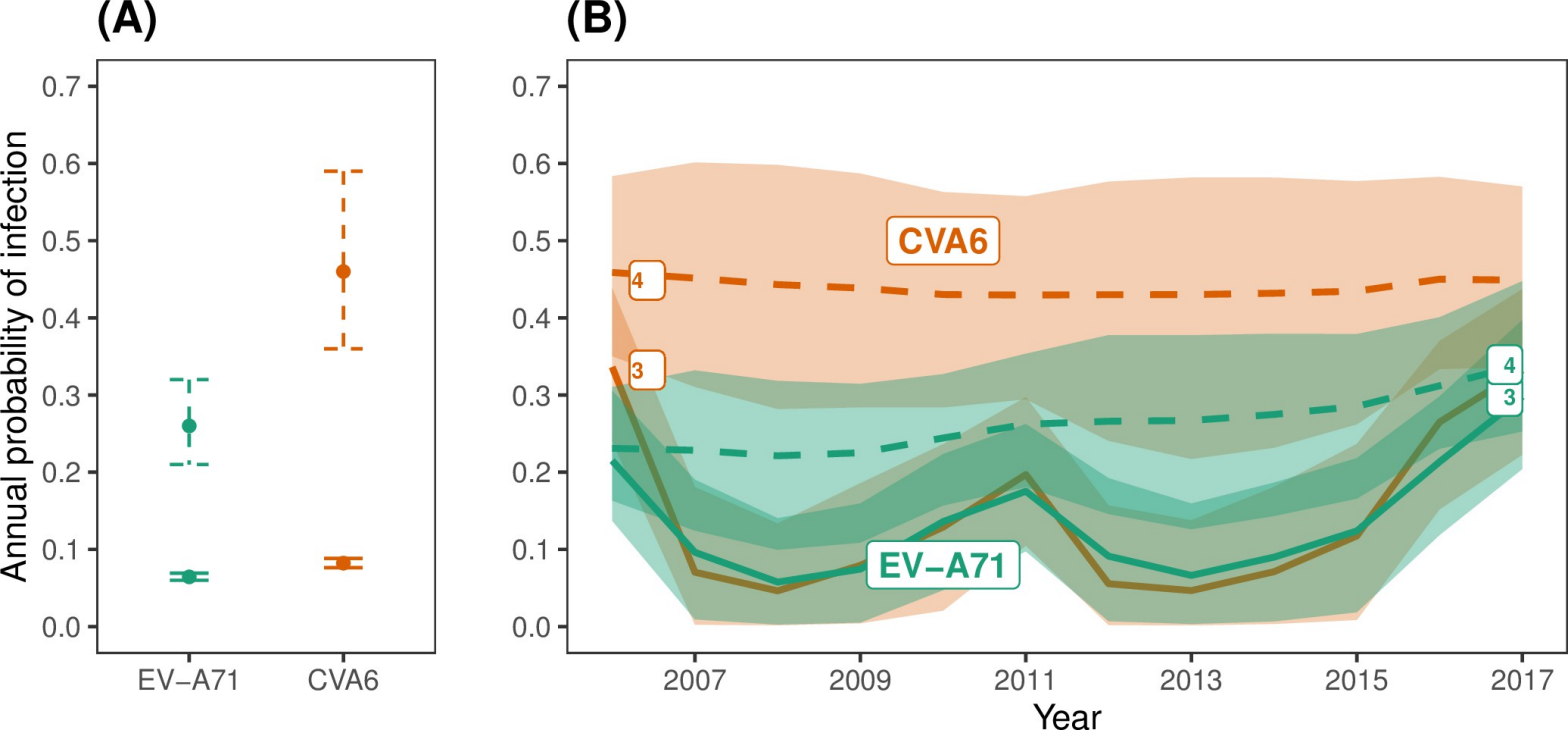
Estimated annual probability of infection for age-constant FOI Models. (A) Estimated annual probabilities of infection for EV-A71 and CVA6 using the age- and time- constant FOI models without seroreversion (Model 1, solid intervals) and with seroreversion (Model 2, dashed intervals). (B) Estimated annual probabilities of infection for EV-A71 and CVA6 using the time-varying FOI models without seroreversion (Model 3, continuous lines), and with seroreversion (Model 4, dashed lines). The lines represent the mean posterior estimates, while the shaded areas represent the 95% credible intervals.

**Fig 4:**
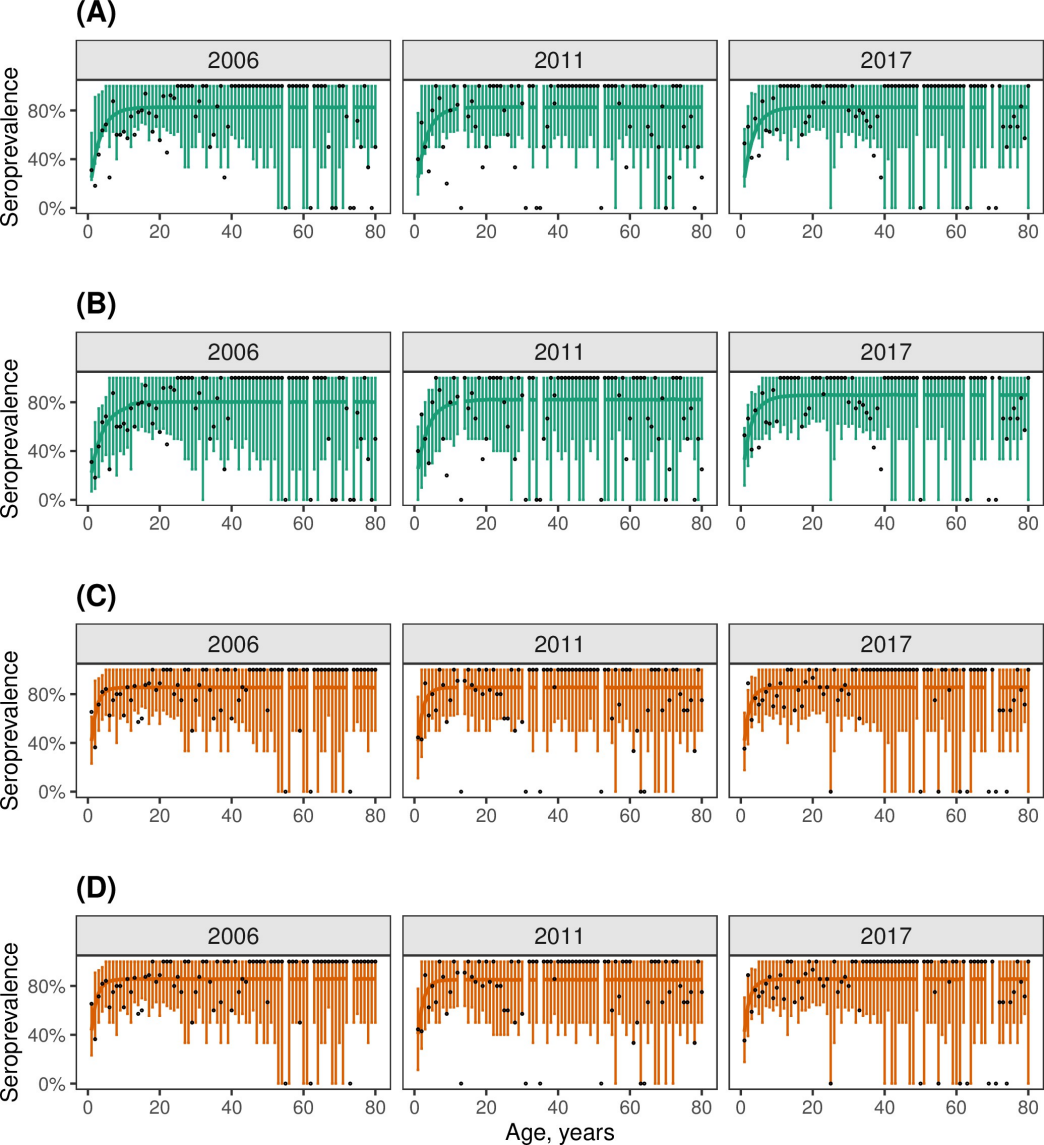
Best age-constant FOI Models (Models 2 and 4) fit to data. (A, C) Posterior predictive check for the age- and time-constant FOI model with seroreversion (Model 2). (B, D) Posterior predictive check for the time-varying FOI model with seroreversion (Model 4). The observed proportion of samples that were seropositive are shown as black circles. The solid lines and error bars represent the model’s mean predicted seropositivity estimates and 95% Bayesian credible intervals for EV-A71 (green) and CVA6 (orange). The gaps in the plots indicate absence of data in the corresponding age(s).

We initially fitted four models that combined a constant or time-varying FOI with or without seroreversion (Models 1–4). For both serotypes, the models without seroreversion (Models 1 and 3), performed poorly (Table 1), as they failed to reproduce a plateau of seroprevalence below 100% in older age classes (S11 and S12 Figs). In contrast, the models with seroreversion (Models 2 and 4) performed similarly well (the difference in ELPD was <4, Table 1), and both estimated such a plateau and provided a good model fit (Fig 4). Under the model with time-constant FOI and seroreversion (Model 2), CVA6 had a higher estimated annual probability of infection than EV-A71 (0.46 (95% CrI, 0.36–0.59) vs. 0.25 (0.21–0.32), respectively) (Fig 3A), and a higher seroreversion rate (0.1 (0.07–0.17) vs. 0.06 (0.04–0.09), respectively) (S3 Table). This resulted in seroprevalence increasing quickly with age for both serotypes and plateauing at 82% from the age of 13 for EV-A71 (Fig 4A) and at 85% from the age of nine for CVA6 (Fig 4C).

**Table 1:** Model comparison and ranking using the LOO-CV method. The ordering in which the models are displayed corresponds to the performance of cross-validation for CVA6.

	Time-varying FOI?	Age-dependent FOI?	Seroreversion?	EV-A71		CVA6	
Model				elpd_diff	se_diff	elpd_diff	se_diff
5	No	Yes	No	-1.1	4.5	0	0
2	No	No	Yes	-4.0	4.5	-2.0	1.6
6	No	Yes	Yes	-1.5	6.3	-2.9	1.4
4	Yes	No	Yes	0	0	-3.4	2.6
3	Yes	No	No	-23.2	11.2	-44.0	12.1
1	No	No	No	-260.4	44.4	-260.9	47.8

Model comparison and ranking of the fitted catalytic models using the approximate leave-one-out cross-validation (LOO-CV) method. LOO-CV calculates the expected log pointwise predictive density (ELPD) for each model, which is a measure of the overall model fit accounting for model complexity. Model ranking is based on the differences in the ELPD and standard error estimates (‘*elpd_diff*’, and ‘*se_diff*’, respectively), where the differences are calculated relative to the model with the largest ELPD.

For the model with time-varying FOI and seroreversion (Model 4), the estimates of the annual probability of infection remained almost stable over the study period for both serotypes (Fig 3): they ranged between 0.21 (0.11–0.29) in 2006 and 0.31 (0.23–0.41) in 2017 for EV-A71; and between 0.46 (0.31–0.60) in 2006 and 0.47 (0.35–0.62) in 2017 for CVA6. This model captured slightly better the increase in seroprevalence by age in the first years of life for both serotypes, than the model with constant FOI and seroreversion (Fig 4).

As expected, the models without seroreversion (Models 1 and 3) estimated lower annual probabilities of infection than the corresponding models with seroreversion (Fig 3), because a loss of seropositivity required more infections to explain the same levels of seroprevalence.

The time-varying FOI model without seroreversion (Model 3) estimated peaks in the FOI in the years of sample collection (2006, 2011 and 2017). We attribute this effect to the way this model accommodates to explain differences in seroprevalence across age, and we think the peaks do not reflect true peaks of transmission in the sampling years.

### A declining FOI with age and/or seroreversion were needed to explain the age profiles of seropositivity

Models 1–4 assumed the same FOI across all ages. However, age and time are intrinsically confounded in seroprevalence measurements, and the estimates of seroreversion could be affected when allowing for differences in the risk of seroconversion with age. Therefore, we further tested two models with a declining FOI with age, one without seroreversion (Model 5) and one with (Model 6).

Model comparison indicated that either age-dependence in the risk of seroconversion or seroreversion were necessary to explain the patterns of variation in seroprevalence with age, although our data characteristics meant we could not differentiate between these two hypotheses, and a model including both seroreversion and age-dependent FOIs fared no better (LOO ELPD estimates of Models 2, 4, 5 and 6 were <4 for both serotypes, and therefore, differences in their performance were considered negligible, Table 1).

**Fig 5:**
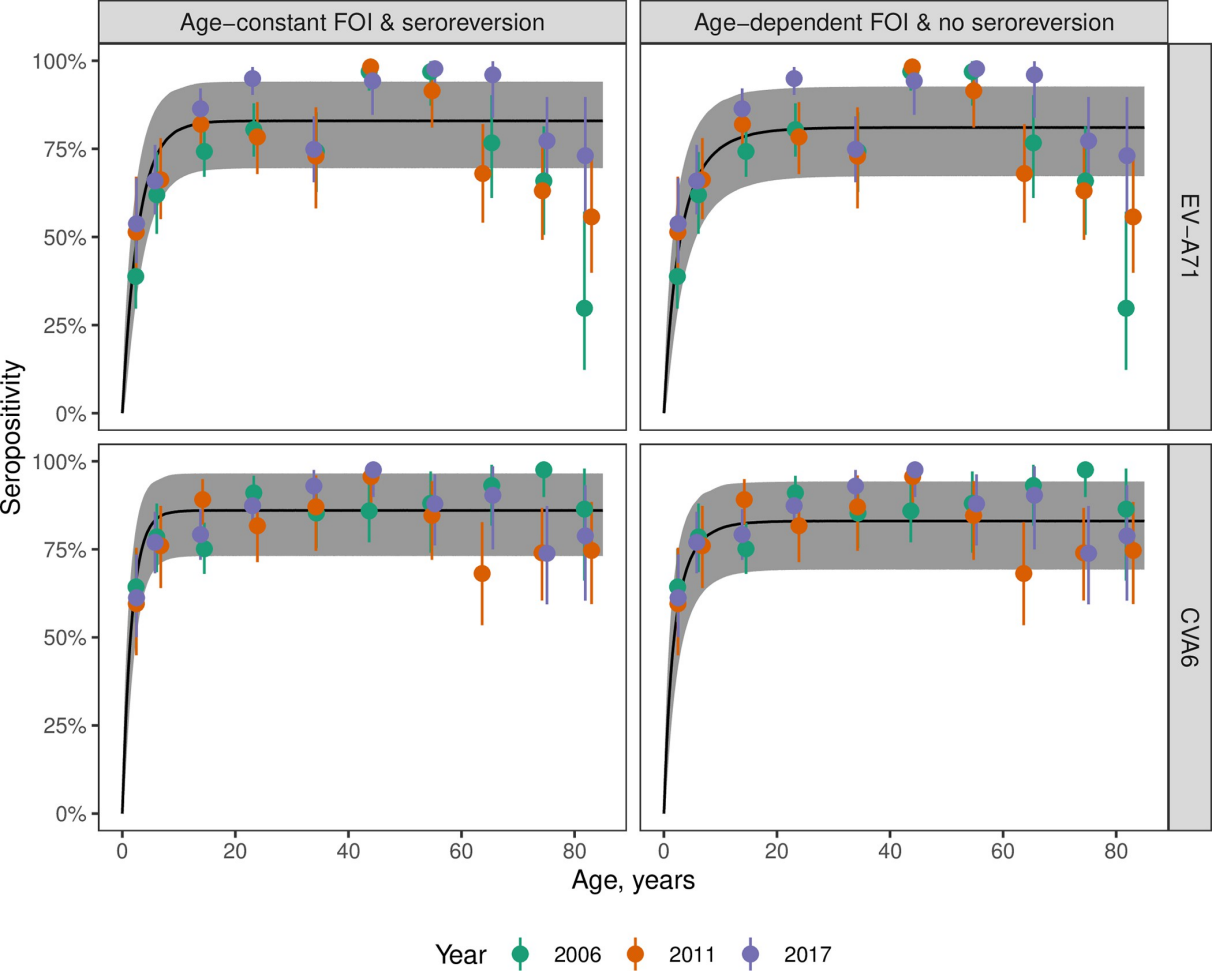
Best time-constant FOI Models (Models 2 and 5) fit to data. The model fits for Model 2 (time- and age-constant FOI with seroreversion) and Model 5 (age-dependent FOI and no seroreversion) are shown for EV-A71 and CVA6. The observed seropositivity values across age are shown as filled circles colored by year of sample collection with 95% binomial confidence intervals. The black line indicates the mean posterior estimate and the shaded region the 95% credible interval.

For both serotypes, the time-constant models with either seroreversion (Model 2) or age-declining FOI (Model 5) provided very similar fits to the data and mirrored the patterns of seroprevalence variation with age (Fig 5): particularly, the rapid increase in seropositivity in the first years of life owing to a high FOI; and the plateauing of seropositivity after the teenage years. For EV-A71, these two models did not explain the decline in seropositivity in older ages, but Model 6, which incorporated both an FOI that declines with age and seroreversion, replicated the pattern of declining seropositivity (S13 Fig). We do not discuss Model 6 further, however, because it provided a similar fit to the data but was more complex than models with either age-dependent FOIs or seroreversion (Table 1).

**Fig 6:**
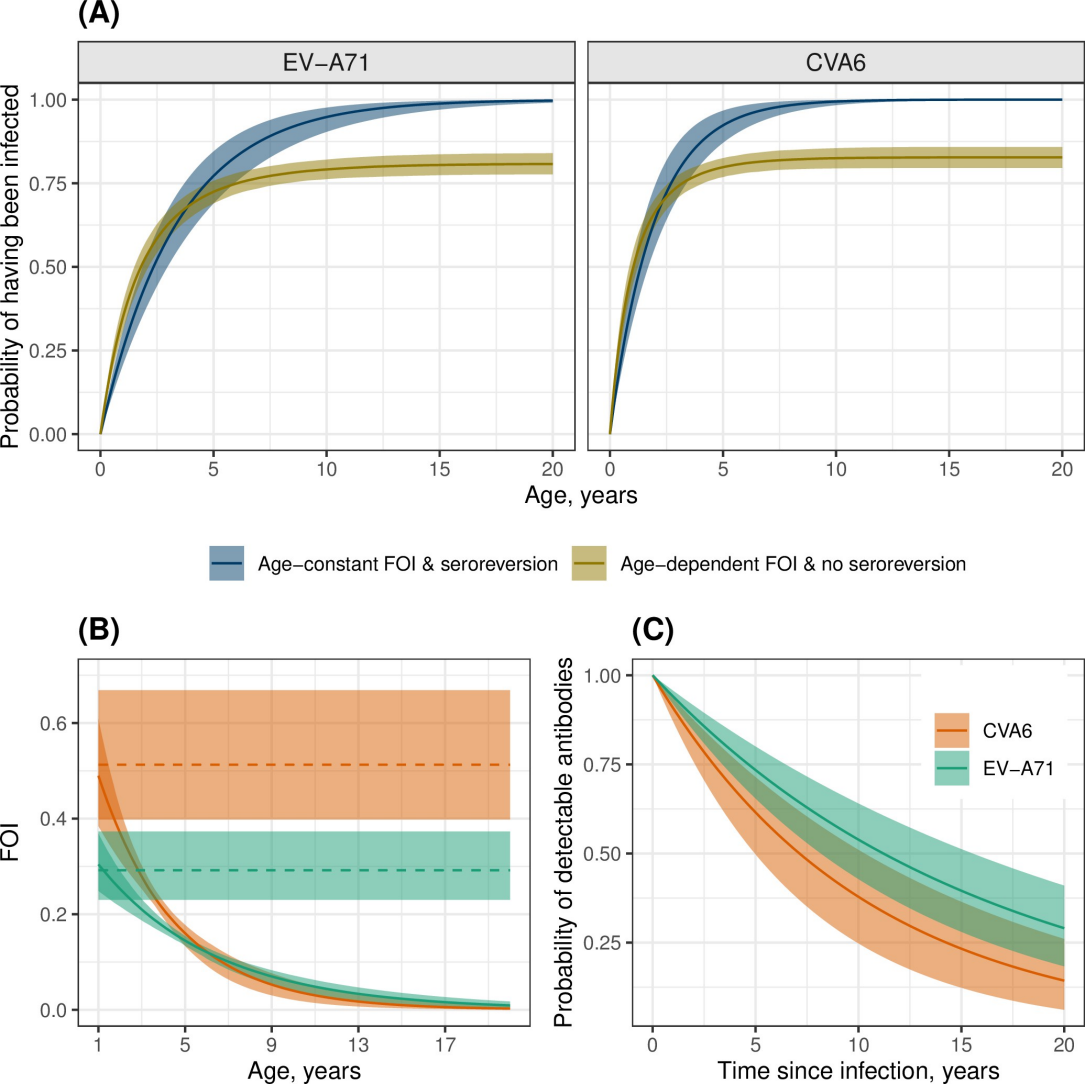
Features of the age- and time-constant FOI model with seroreversion (Model 2) and the age-dependent FOI model without seroreversion (Model 5). (A) Estimated probabilities of at least one infection for EV-A71 and CVA6 by age for the models with age- and time-constant FOI and seroreversion (Model 2) and age-dependent FOI and no seroreversion (Model 5). The solid line indicates the mean posterior estimate, and the shaded region represents the 95% credible interval. (B) Estimated age-dependent FOI using Model 5 (curved line) and age-constant FOI using Model 2 (straight line). (C) Estimated probability of detecting serum neutralizing antibodies since the time of seroconversion for EV-A71 and CVA6 using the model with time-constant FOI and seroreversion (Model 2).

To explore differences in the dynamics of seroconversion under the model with time-constant FOI and seroreversion (Model 2) and the model with age-dependent FOI but no seroreversion (Model 5), we estimated the probability that an individual has been infected by each serotype at a given age (Fig 6A). This probability was greater for CVA6 than EV-A71, particularly in younger ages. The model with an FOI that declines with age and no seroreversion (Model 5) predicted that some individuals never become infected during their lifetimes (20%, 95% CrI, 17% - 23% for EV-A71; 18%, 95% CrI, 15% - 21% for CVA6), whereas the model with time-constant FOI and seroreversion (Model 2) indicated that, if an individual lives long enough, they will have been infected by each serotype (Fig 6A), reaching a probability of 95% of having been infected at least once with CVA6 by the age of 5.9 years and at least once with EV-A71 by the age of 10.3 years (Fig 6A).

In the age-dependent model without seroreversion (Model 5), the FOI fell sharply with age, and the rate of decline was higher for CVA6 than EV-A71 (Fig 6B), with our estimates suggesting that an overwhelming proportion of all first infections occur in individuals under 5 years (CVA6: 96.8% (95% CrI, 96.2% - 97.5%); EV-A71: 90% (89.6% - 92%)). The corresponding results from the time-constant model with seroreversion (Model 2), whilst lower, also indicate that the bulk of infections occur in those under 5 years of age (CVA6: 92% (95% CrI, 86.8% - 97%); EV-A71: 77% (69.5% - 84.8%)).

In the time-constant model with seroreversion (Model 2), the rate of decline of antibody detectability was faster for CVA6 than EV-A71, corresponding to a shorter duration of antibody detectability after seroconversion (10 years (7–15) vs. 16 years (12–25), respectively) (S3 Table, Fig 6C). For both serotypes, the probability of detecting antibodies 12 years after infection was estimated at less than half.

We performed a range of prior sensitivity analyses across the models (S1 Text), and the estimates were largely unchanged (S4, S5, and S6 Tables) meaning our conclusions above remained the same.

A sensitivity analysis to the accuracy of the assay also unchanged the main conclusions. The three scenarios tested assuming imperfect accuracy of the serology assay (1: Se = 0.9, Sp = 1.0; 2: Se = 0.85, Sp = 1.0; 3: Se = 0.9, Sp = 0.9) provided estimates of the annual probability of infection similar to those obtained in the main analysis assuming 100% accuracy, except for the simplest model, with constant FOI and no seroreversion (Model 1) (S14 and S15 Figs). In the sensitivity analysis, the duration of seropositivity (Models 2, 4 and 6) was generally longer (S7 and S8 Tables), and the decline of the FOI with age (Models 5 and 6), slower (S8 Table), compared to the main analysis. Model comparison provided similar results across the three scenarios for each serotype (S9 Table). However, interestingly, the differences in model performance were smaller across the six models for decreasing sensitivity of the assay.

## Discussion

Enterovirus infections, although can cause serious illnesses such as neurological diseases (often as a result of complications of mild infections), are often underdiagnosed and underreported in the absence of routine surveillance at the community level. This leads to underestimation of pathogen abundance and circulation. In England, EV-A71 and CVA6 detections reported by health authorities over 2006–2017 show an apparent increase in disease cases, that is clearer for CVA6 than EV-A71 (Fig 1). However, it is unclear to what extent this increase in virus detections was driven by an increase in virus transmission. Here, we infer EV-A71 and CVA6 transmission intensity in England during 2006–2017 by fitting catalytic models to seroprevalence data against contemporary strains of EV-A71 and CVA6 [31].

For both serotypes, our data and models do not support significant changes in transmission intensity over the study period (2006–2017). As these results do not support an increase in transmission, the alternative explanation for the observed increase in CVA6 detections in England in 2006–2017 is a change in virus pathogenicity (i.e., a higher probability of an infection to result in disease). These results are in agreement with a modelling study of sentinel surveillance data from Japan [8]. CVA6 started to cause large outbreaks of HFMD in Japan in 2011, after years of smaller outbreaks, mostly associated with herpangina rather than HFMD. The modelling study tested different hypotheses that could explain the sudden occurrence of large CVA6-related HFMD outbreaks from 2011, and found that an increase in pathogenicity, but not changes in transmission, explained the Japanese data best [8].

Although not recorded in the UK, acute EV-A71 infection waves occurred in Austria, France, Germany, and the Netherlands in the 2000–2010 decade [40], and EV-A71 outbreaks of severe neurological diseases occurred in neighboring European countries in the late 2010’s, including Spain in 2016 (12) and Germany in 2019 (13), and the USA (Colorado) in 2018 (14). Our results do not support the hypothesis that these outbreaks were due to increased transmission of EV-A71, but the analysis of seroprevalence data from the countries affected would allow to corroborate this.

Models which included seroreversion (i.e., loss of detectable antibodies) or a declining FOI with age were favored in the model comparison. In reality, both mechanisms may be present, with a declining FOI with age perhaps reflecting acquisition of protection or a reduction of risk contacts with age. However, an important knowledge gap in the epidemiology of enteroviruses is how often re-infections with the same enterovirus serotype occur, and whether seropositive individuals are fully protected against infection. A study from China on the recurrence of HFMD episodes reported re-infections with the same serotype, but with probabilities lower than recurrences with other serotypes [41] suggesting a certain degree of serotype-specific protection, at least against HFMD symptoms.

Here, the model with time-constant FOI and seroreversion estimated a duration of antibody detection of 16 (12–25) years for EV-A71 and 10 (7–15) years for CVA6. However, there are no substantial data or empirical estimates of duration of neutralizing antibodies for EV-A71 or CVA6 available to compare, and the only data that comes close is from a recent study which showed that neutralizing antibody titers for EV-A71 and CVA16 remain high, years after illness onset [42]. However, CVA6 was not tested in that study and the longest sampling time was 26 months after illness onset [42]. A firm picture of the kinetics of antibody responses in enterovirus infections is currently lacking.

Despite the different pattern on the number of EV-A71 and CVA6 detections reported over time in England, the analysis of seroprevalence data suggests strong similarities in the transmission dynamics of these two serotypes during the study period. This probably reflects shared transmission routes. Interestingly, the best models with seroreversion (Models 2 and 6) estimate a longer duration of seropositivity following infection for EV-A71 than for CVA6, as well as a lower FOI for EV-A71 than for CVA6. This suggests that reinfections with CVA6 occur more frequently than with EV-A71.

With data at only three time points, we were only able to estimate general trends in the force of infection over several years. Inference of possible transmission changes at a thinner timescale would require more precise age-specific seroprevalence estimates. Prioritizing sampling younger ages over older ones, may be a strategy to maximize the information that will support the estimates of the force of infection. Similarly, sampling more often, may allow to infer transmission changes at a thinner timescale. This could be particularly important for EV-A71, which exhibited a 2–3-year cycle in the virus detection data.

Other limitations are inherent to our study. We assumed complete single-serotype neutralizing antibody reactivity, but we cannot discount potential non-specific serologic cross-reactivity arising from varied exposure histories with other enteroviruses. Presence of overlapping viral receptor repertoires [43,44] and cross-serotype immunological interactions between enteroviruses has been documented [45]. However, these factors are unlikely to bias our results since virus neutralization tests, as used in our study, are considered the most sensitive and specific assays for detecting virus-specific neutralizing antibodies, and any cross-neutralization is thought to be very limited or absent. A future direction for this work is to develop a model framework that uses antibody titers, instead of classifying individuals either as seropositive or seronegative based on a cut-off, which leads to a loss of information.

Our analysis provides an improved picture of EV-A71 and CVA6 transmission in England and may be useful for estimation of disease burden and severity of infections caused by these viruses. It further underscores the value of serological data to unravel transmission patterns, particularly for pathogens that cause many asymptomatic infections and diseases for which surveillance is passive. The interpretation of the number of the reported infections from the passive surveillance systems may lead to erroneous hypothesis about the levels of pathogen circulation, as has been demonstrated here. This work contributes to our understanding of the causes underlying the emergence of enterovirus related disease outbreaks and supports the hypothesis of phenotypic changes as a driver of enterovirus emergence. There is a need for further virology studies of EV-A71 and CVA6 to investigate the potential mechanisms of evolution of phenotype and pathogenicity including the genetic determinants of replication, tropism and receptor usage that have contributed to the emergence of associated disease outbreaks. Such investigations would include identification of clones of contemporary strains based on evolutionary trees, followed by detailed mapping or characterization of phenotypic effects associated with key genetic mutations.

## Supporting information

S1 Text Additional methods. Detailed description of models and sensitivity analyses.
(DOCX)

S1 Fig Prior predictive simulations. Prior predictive simulations of the age-profile of seropositivity to assess the appropriateness of priors of parameters ρ and λ used in the constant FOI models (Model 1—panels A and B, and Model 2—panels C and D). That is, the figures show the implications of a prior in terms of what it says the data is going to look like. Panels A and B shows the simulated seropositivity using *exponential(1)* and *exponential(10)* on λ, respectively. Panel C shows simulations using *exponential(1)* on λ and *exponential(20)* on ρ, while panel D shows the simulated seropositivity using *exponential(10)* on λ and *exponential(20)* on ρ. The shaded area is the 95% interval and the solid line is the mean estimate of seropositivity.
(TIF)

S2 Fig Prior predictive simulations. Prior predictive simulations of the age-profile of seropositivity to assess the appropriateness of priors of parameters *β*, ρ and λ used in the age-dependent FOI models (Models 5—panels A and B, and Model 6—panels C and D). That is, the figures show the implications of a prior in terms of what it says the data is going to look like. Panels A shows the simulated seropositivity using *exponential(1)* on λ and *exponential(20)* on *β*; while panel B shows the simulated seropositivity using *exponential(10)* on λ and *exponential(20)* on *β*. Panel C shows simulations using *exponential(1)* on λ, *exponential(20)* on *β* and *exponential(20)* on ρ; while panel D shows simulations using *exponential(10)* on λ, *exponential(20)* on *β* and *exponential(20)* on ρ. The shaded area is the 95% interval, and the solid line is the mean estimate of seropositivity.
(TIF)

S3 Fig Total enterovirus detections. Total number of genotyped enterovirus-positive referrals from England, UK, submitted for genotyping to UKHSA from 2006 to 2017.
(TIF)

S4 Fig Enterovirus D68 (EV-D68) and Coxsackievirus A16 (CVA16) detections among all genotyped enterovirus-positive referrals in England, UK, between 2006 and 2017. Contribution of EV-D68 (A) and CVA16 (B) to the overall genotyped enterovirus-positive referrals submitted for genotyping to UKHSA each year from 2006 to 2017.
(TIF)

S5 Fig Geographical distribution of the number of genotyped enterovirus-positive referrals from England, UK, submitted for genotyping to UKHSA, from 2006 to 2017. Only data for cities with over 90 detections during the 12-year period are shown.
(TIF)

S6 Fig Geographical distribution of the proportion of genotyped enterovirus-positive referrals from England, UK, submitted for genotyping to UKHSA, from 2006 to 2017. Only data for cities with over 90 detections during the 12-year period are shown.
(TIF)

S7 Fig Availability of information on sample sources over time. Total number (A) and proportion (B) of genotyped enterovirus-positive referrals from England, UK, submitted for genotyping to UKHSA, from 2006 to 2017, with and without information on sample sources.
(TIF)

S8 Fig Evolution of sample sources submitted for enterovirus genotyping. Sample sources recorded from genotyped enterovirus-positive referrals from England, UK, submitted for genotyping to UKHSA from 2006 to 2017. Only data for the 6 most frequently reported sample sources during 2006–2017 are shown.
(TIF)

S9 Fig Proportion of skin swabs positive for Coxsackievirus A6 over time. Total number (A) and proportion (B) of skin swabs positive for CVA6 each year among those referred from England, UK, submitted for genotyping to UKHSA from 2006 to 2017.
(TIF)

S10 Fig Antibody titer distributions for each cross-sectional serosurvey. Antibody titer distributions for the three cross-sectional serosurveys (2006, 2011 and 2017) for EV-A71 (A) and CVA6 (B), and for the three serosurveys combined, but restricted to the younger population of 1–10 years old (C), for EV-A71 (C, left) and for CVA6 (C, right).
(TIF)

S11 Fig Model 1 fit to data. Model 1 (constant force of infection and no seroreversion) fit to data for EV-A71 (A) and CVA6 (B). The observed proportion of samples that were seropositive are shown as black circles. The solid lines and shaded area represent the model’s mean predicted seropositivity estimates and 95% Bayesian Credible Intervals. The gaps in the plots indicate absence of data in the corresponding age(s).
(TIF)

S12 Fig Model 3 fit to data. Model 3 (time-varying FOI model) fit to data for EV-A71 and CVA6. The observed proportion of samples that were seropositive are shown as black circles. The solid lines and shaded area represent the model’s mean predicted seropositivity estimates and 95% Bayesian Credible Intervals. The gaps in the plots indicate absence of data in the corresponding age(s).
(TIF)

S13 Fig Model 6 fit to data. Model 6 (age-dependent force of infection and seroreversion) fit to data for EV-A71 (A) and CVA6 (B). The observed proportion of samples that were seropositive are shown as black circles. The solid lines and shaded area represent the model’s mean predicted seropositivity estimates and 95% Bayesian Credible Intervals. The gaps in the plots indicate absence of data in the corresponding age(s).
(TIF)

S14 Fig Sensitivity analysis to serology assay accuracy for Models 1 to 4. Estimates of the annual probability of infection for different values of sensitivity and specificity of the assay: 0, Se = 100%, Sp = 100% (results presented in the main text); 1, Se = 90%, Sp = 100%; 2, Se = 85%, Sp = 100%; and 3, Se = 90%, Sp = 90%. The corresponding parameter estimates for the seroreversion rate (ρ) for Models 2 and 4 are shown in S7 Table.
(TIF)

S15 Fig Sensitivity analysis to serology assay accuracy for Models 5 and 6. Estimates of the annual probability of infection at age 1 for different values of sensitivity and specificity of the assay: 0, Se = 100%, Sp = 100% (results presented in the main text); 1, Se = 90%, Sp = 100%; 2, Se = 85%, Sp = 100%; and 3, Se = 90%, Sp = 90%. The corresponding parameter estimates for β and ρ are listed in S8 Table.
(TIF)

S1 Table Description of the different catalytic models and priors used. (DOCX)

S2 Table Description of the serology dataset. (DOCX)

S3 Table Parameter estimates of the catalytic models with constant FOI over time. (DOCX)

S4 Table Parameter estimates resulting from sensitivity analyses on the λ prior. (DOCX)

S5 Table Parameter estimates resulting from sensitivity analyses on the *ρ* prior for EV-A71. (DOCX)

S6 Table Parameter estimates resulting from sensitivity analyses on the *ρ* prior for CVA6. (DOCX)

S7 Table Parameter estimates for ρ resulting from sensitivity analyses to serology assay accuracy for Models 2 and 4. Sensitivity (Se) and specificity (Sp) values were labelled as: 0, Se = 100%, Sp = 100% (results presented in the main text); 1, Se = 90%, Sp = 100%; 2, Se = 85%, Sp = 100%; and 3, Se = 90%, Sp = 90%.
(DOCX)

S8 Table Parameter estimates (ρ and β) resulting from sensitivity analyses to serology assay accuracy for Models 5 (age-dependent FOI without seroreversion) and 6 (age-dependent FOI with seroreversion). Sensitivity (Se) and specificity (Sp) values were labelled as: 0, Se = 100%, Sp = 100% (results presented in the main text); 1, Se = 90%, Sp = 100%; 2, Se = 85%, Sp = 100%; and 3, Se = 90%, Sp = 90%.
(DOCX)

S9 Table Model comparison and ranking using the LOO-CV method. Models are compared and ranked across assay accuracy parameters: sensitivity (Se) and specificity (Sp).
(DOCX)
