# Supplementary material for: Enterovirus A71 and coxsackievirus A6 circulation in England, UK, 2006–2017: A mathematical modelling study using cross-sectional seroprevalence data
Source: PLoS Pathog. 2024 Nov 20;20(11):e1012703. doi: 10.1371/journal.ppat.1012703 (PMC11578500; doi:10.1371/journal.ppat.1012703)
Supplement: S1 Text — Detailed description of models and sensitivity analyses. (DOCX) [file ppat.1012703.s001.docx]

**SUPPORTING INFORMATION**

Enterovirus A71 and coxsackievirus A6 circulation in England, UK, 2006 – 2017: a mathematical modelling study using cross-sectional seroprevalence data

Everlyn Kamau^1*^, Ben Lambert^2^, David J Allen^3^, Cristina Celma^4^, Stuart Beard^4^, Heli Harvala^5,6^, Peter Simmonds^1^, Nicholas C. Grassly^7^, Margarita Pons-Salort^7*^

^1^Nuffield Department of Medicine, University of Oxford, Oxford, UK

^2^Department of Mathematics, College of Engineering, Mathematics and Physical Sciences, University of Exeter, Exeter, UK

^3^Department of Comparative Biomedical Sciences, Section Infection and Immunity, School of Veterinary Medicine, Faculty of Health and Medical Sciences, University of Surrey, Guildford, UK

^4^Enteric Virus Unit, UK Health Security Agency, Colindale, London, UK

^5^Microbiology Services, NHS Blood Transfusion, London, UK

^6^Infection and Immunity, University College of London, London, UK

^7^MRC Center for Global Infectious Disease Analysis, School of Public Health, Imperial College London, London, UK

*** Corresponding author: [everlyn.kamau@ucsf.edu](mailto:everlyn.kamau@ucsf.edu), [m.pons-salort@imperial.ac.uk](mailto:m.pons-salort@imperial.ac.uk)

**Catalytic models**

**Model 1**:

Model 1 assumes a constant force of infection (*λ*) over time and no seroreversion. The dynamics are described by the differential equation:

$$z’\left( a \right)= \lambda-\lambda z(a)$$

λ

Seropositive (Z(a))

Seronegative (X(a))

**Model 2**:

Model 2 assumes a constant force of infection (*λ*) over time and seroreversion, with seroreversion rate ρ. The dynamics are described by the differential equation:

$$z’\left( a \right)= \lambda-( +)z(a)$$

λ

Seronegative (X(a))

Seropositive (Z(a))

ρ

**Model 3:**

Model 3 assumes a time-varying FOI without seroreversion. Let’s denote the sampling year by *S*. For an individual of age *a_j_* in year *S*, the probability of being seropositive will depend on the FOI during the years between when they were born, *S−a_j_*, and the sampling year, *S*, which we denote by $\lambda_{S-a_{j}}$*, …, λ_S−1_, λ_S_*. We assume individuals cannot be infected the year they are born. The probability for a 1-year-old being seronegative in year *S* is given by:

$$P_{\_(a_{1,S})}=e^{-\left( \lambda_{S} \right)}$$

For an individual of age *a_j_*, the probability to be seronegative in year *S* is calculated from the cumulative risk of infection from the year the individual was age one *S−a_j_+1* to the sampling year *S:*

$$P_{{\_}_{(a_{j,S})}}= exp(-\sum_{{t=S-a_{j}+1}}^{S} \lambda_{t})$$

**Model 4:**

Model 4 assumes a time-varying FOI with seroreversion. Let’s denote by *S* the sampling year. For an individual of age *a_j_* in year *S*, the probability of being seropositive will depend on the FOI during the years between when they were born, *S−a_j_*, and the sampling year, *S*, which we denote by $\lambda_{S-a_{j}}$*, ..., λ_S−1_, λ_S_*. We denote the rate of seroreversion by *ρ*, and we assume individuals cannot be infected the year they are born. Here, the probability for a 1-year-old being seronegative in year *S* is given by:

$$P_{\_(a_{1,S})}=e^{-\left( \lambda_{S}+\rho\right)}+\frac{\rho}{\lambda_{S}+\rho}\left( 1-e^{-\left( \lambda_{S}+\rho\right)} \right)$$

where the first term is the probability of not getting infected and the second one is the probability of getting infected and sero-reverting. We used the derivations by Hozé et al. ([1](#_ENREF_1)) to get the probability for an individual aged *a_j_* to be seronegative in sampling year *S*, which can be obtained by recursion:

$$P_{{\_}_{(a_{j,S})}}={{P_{\_}}_{\left( a_{j-1,S-1} \right)}\times e}^{-\left( \lambda_{S}+\rho\right)}+\frac{\rho}{\lambda_{S}+\rho}\left( 1-e^{-\left( \lambda_{S}+\rho\right)} \right)$$

**Models 5 and 6: Age-dependent FOI models**

We modified Models 1 and 2 to assume that the force of infection follows an exponential relationship with age, i.e., FOI decays exponentially by age:

$$\lambda\left( a \right)=\lambda_{1}e^{-\beta(a-1)}.$$

This resulted in Model 5, which allowed no seroreversion, where the seropositivity at age $a$ is given by ([2](#_ENREF_2)):

$$z\left( a \right)=1-exp(-\int_{1}^{a} \lambda\left( a^{'} \right)da^{'}).$$

This expression implies that, if $\lambda_{1}>0$ and $\beta>0$, seropositivity increases with age and plateaus at: $z\left( \infty\right)=1-exp(\frac{\lambda_{1}}{\beta})$.

For the model including seroreversion, Model 6, the corresponding quantity is given by ([2](#_ENREF_2)):

$$z\left( a \right)=1-exp(-\int_{1}^{a} \lambda\left( a^{'} \right)da^{'})-\rho\int_{1}^{a} exp[-\int_{a^{'}}^{a} \left( \lambda\left( a \right)+\rho\right)da^{''})] da^{'} ,$$

This model implies that, if $\lambda_{1}>0$, $\beta>0$ and $\rho>0$, that seropositivity reaches a peak before declining towards $z\left( \infty\right)=0$. Intuitively, this is because older age groups lose antibody detectability without it being replenished by infection.

**Sensitivity analyses**

***Sensitivity analyses to the priors***

We explored how FOI estimates from Models 1 and 2 changed as we assumed a different prior distribution ($\lambda\sim exponential\left( 10 \right)$ instead of $exponential\left( 1 \right)$) that would allow for lower seropositivity values. Using the alternative prior in Model 1, the mean annual probability of infection was estimated at 0.062 (95% Credible Interval, 0.058–0.067) and 0.079 (0.073-0.084), for EV-A71 and CVA6, respectively. With Model 2, the mean annual probability of infection was estimated at 0.25 (0.2–0.3) and 0.4 (0.33-0.49) for EV-A71 and CVA6, respectively; and a mean duration of seropositivity of 16 (12-23) and 11 (8-16) years for EV-A71 and CVA6, respectively. For Model 1, both the initial and alternative prior distributions for $\lambda$ resulted in similar model fits to the seroprevalence data (i.e., similar posterior predictive checks), which is shown in Fig S11.

We also explored how parameter estimates from Models 2, 4 and 6 changed if we assumed a shorter duration of seropositivity by changing the prior distribution for seroreversion rate to $\rho\sim exponential\left( 1 \right)$, corresponding to a typical duration of seropositivity of 1 year (instead of $\rho\sim exponential\left( 20 \right)$). Using the alternative prior yielded very similar estimates for the three models (S5 and S6 Tables).

***Sensitivity analyses to assay performance***

We conducted a sensitivity analysis to explore the impact of potential imperfect accuracy of the serology assay. To account for imperfect sensitivity (*Se*) and specificity (*Sp*), we modified the likelihood as follows:

$$p\left( a \right)\sim\text{Binomial}\left( n\left( a \right), Se*z\left( a \right)+\left( 1-Sp \right)*(1-z\left( a \right)) \right)$$

where *Se* and *Sp* are fixed and take values between 0 and 1. Standardized protocols for enterovirus microneutralization assays do not exist yet ([3](#_ENREF_3)) meaning these are in-house assays, for which estimates of sensitivity and specificity are not available. As microneutralization assays for enteroviruses are considered highly specific, we tested three scenarios where we considered specificity to be non-inferior to sensitivity: 1, Se=90%, Sp=100%; 2, Se=85%, Sp=100%; and 3, Se=90%, Sp=90%.

**REFERENCES**

1. Hoze N. RSero [Available from: <https://htmlpreview.github.io/?https://github.com/nathoze/Rsero/blob/master/vignettes/models.html>.

2. Imai N, Dorigatti I, Cauchemez S, Ferguson NM. Estimating dengue transmission intensity from sero-prevalence surveys in multiple countries. PLoS Negl Trop Dis. 2015;9(4):e0003719.

3. Coudere K, Benschop KSM, Koen G, van Eijk H, Harvala H, Bailly JL, et al. Assessment of twelve echovirus virus-neutralisation assays in Europe: recommendations for harmonisation of non-polio enterovirus sero-surveillance studies. J Gen Virol. 2024;105(9).
