# Supplementary figures and images for: Enterovirus A71 and coxsackievirus A6 circulation in England, UK, 2006–2017: A mathematical modelling study using cross-sectional seroprevalence data
Source: PLoS Pathog. 2024 Nov 20;20(11):e1012703. doi: 10.1371/journal.ppat.1012703 (PMC11578500; doi:10.1371/journal.ppat.1012703)

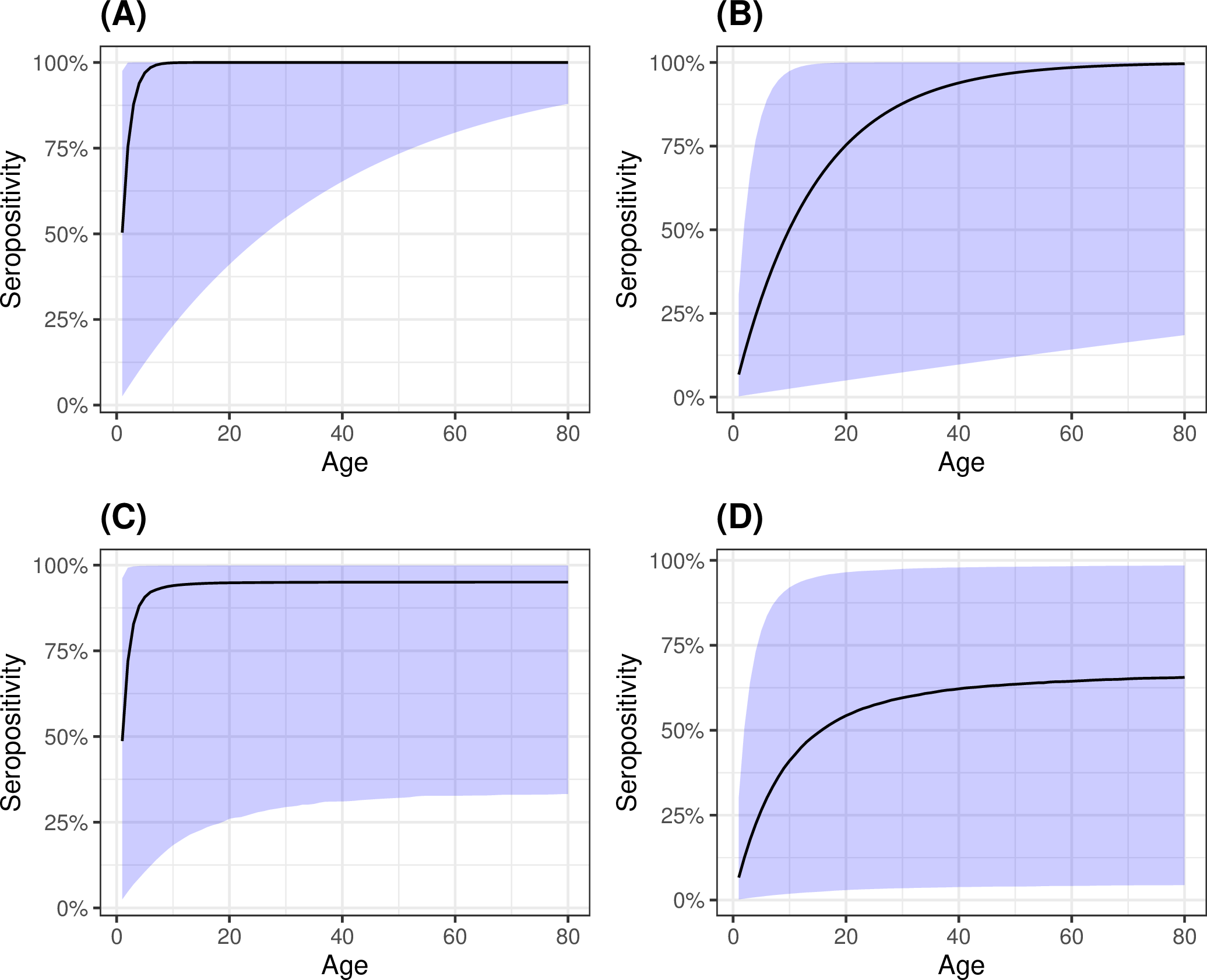

Supplement: S1 Fig — Prior predictive simulations of the age-profile of seropositivity to assess the appropriateness of priors of parameters ρ and λ used in the constant FOI models (Model 1—panels A and B, and Model 2—panels C and D). That is, the figures show the implications of a prior in terms of what it says the data is going to look like. Panels A and B shows the simulated seropositivity using exponential(1) and exponential(10) on λ, respectively. Panel C shows simulations using exponential(1) on λ and exponential(20) on ρ, while panel D shows the simulated seropositivity using exponential(10) on λ and exponential(20) on ρ. The shaded area is the 95% interval and the solid line is the mean estimate of seropositivity. (TIF) [file ppat.1012703.s002.tif]

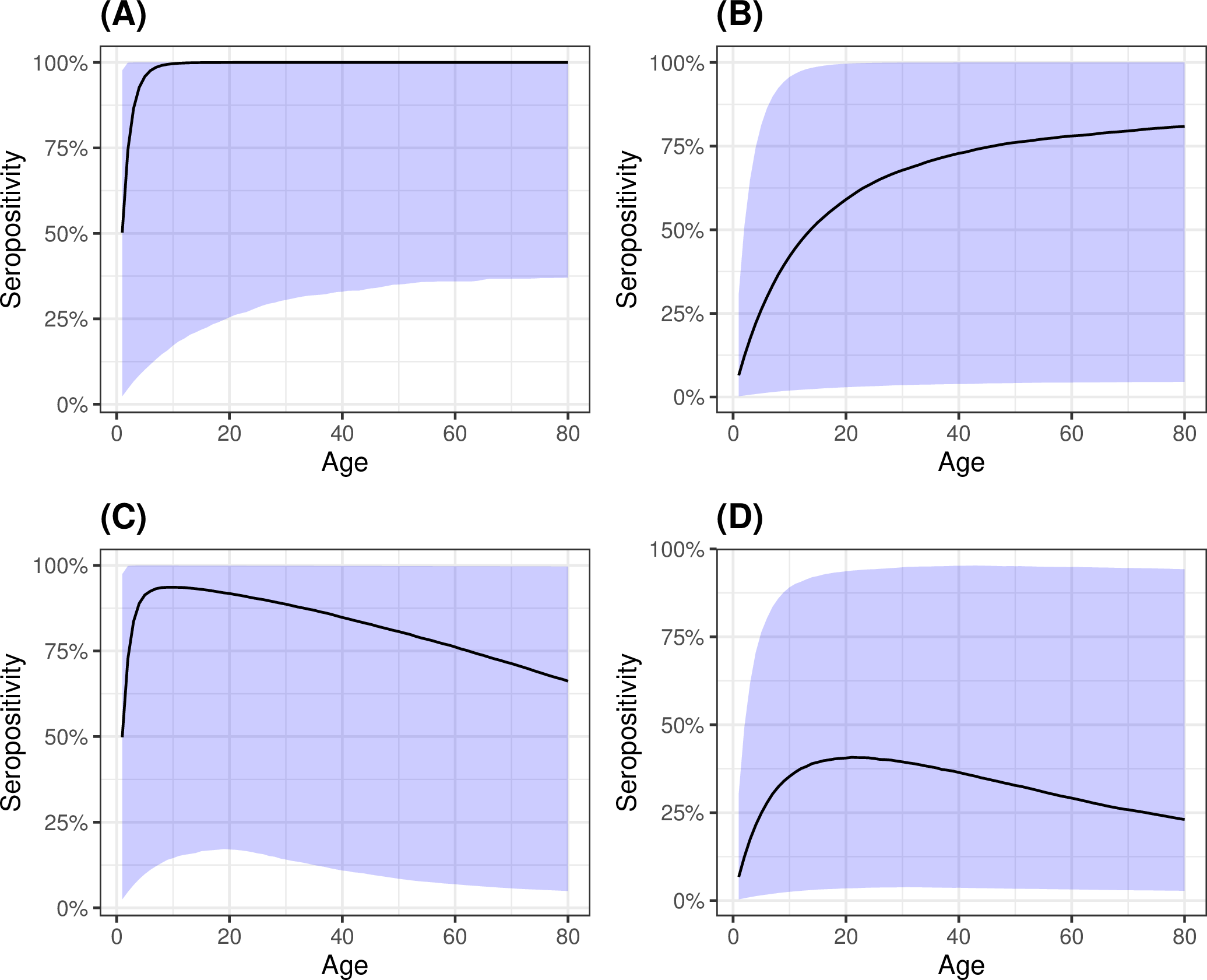

Supplement: S2 Fig — Prior predictive simulations of the age-profile of seropositivity to assess the appropriateness of priors of parameters β, ρ and λ used in the age-dependent FOI models (Models 5—panels A and B, and Model 6—panels C and D). That is, the figures show the implications of a prior in terms of what it says the data is going to look like. Panels A shows the simulated seropositivity using exponential(1) on λ and exponential(20) on β; while panel B shows the simulated seropositivity using exponential(10) on λ and exponential(20) on β. Panel C shows simulations using exponential(1) on λ, exponential(20) on β and exponential(20) on ρ; while panel D shows simulations using exponential(10) on λ, exponential(20) on β and exponential(20) on ρ. The shaded area is the 95% interval, and the solid line is the mean estimate of seropositivity. (TIF) [file ppat.1012703.s003.tif]

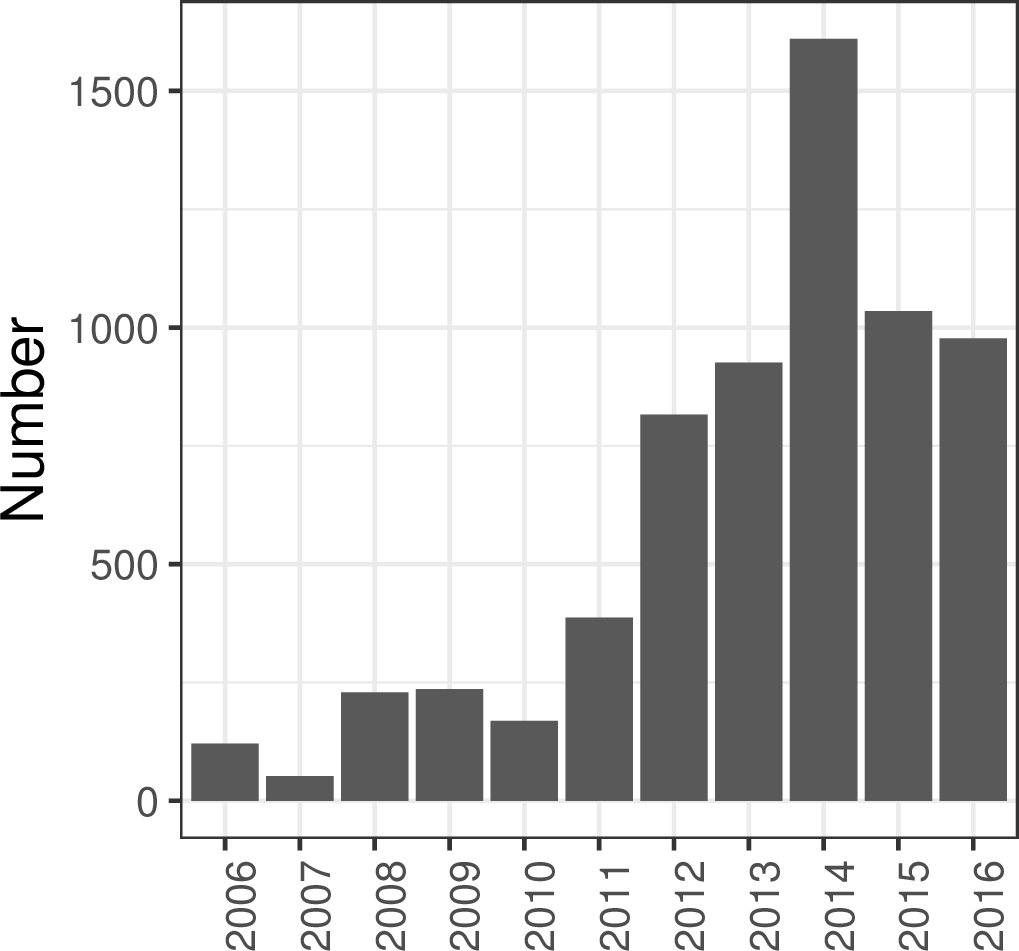

Supplement: S3 Fig — Total number of genotyped enterovirus-positive referrals from England, UK, submitted for genotyping to UKHSA from 2006 to 2017. (TIF) [file ppat.1012703.s004.tif]

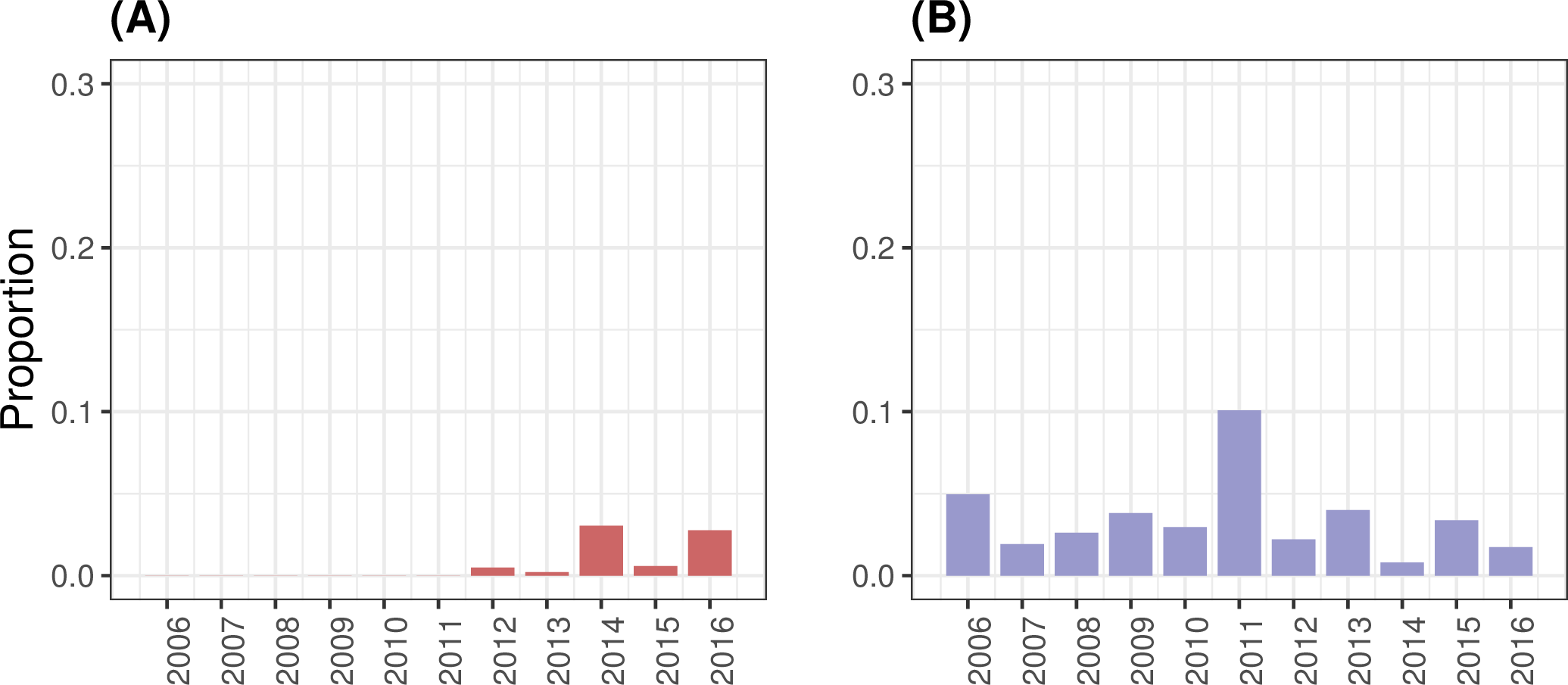

Supplement: S4 Fig — Contribution of EV-D68 (A) and CVA16 (B) to the overall genotyped enterovirus-positive referrals submitted for genotyping to UKHSA each year from 2006 to 2017. (TIF) [file ppat.1012703.s005.tif]

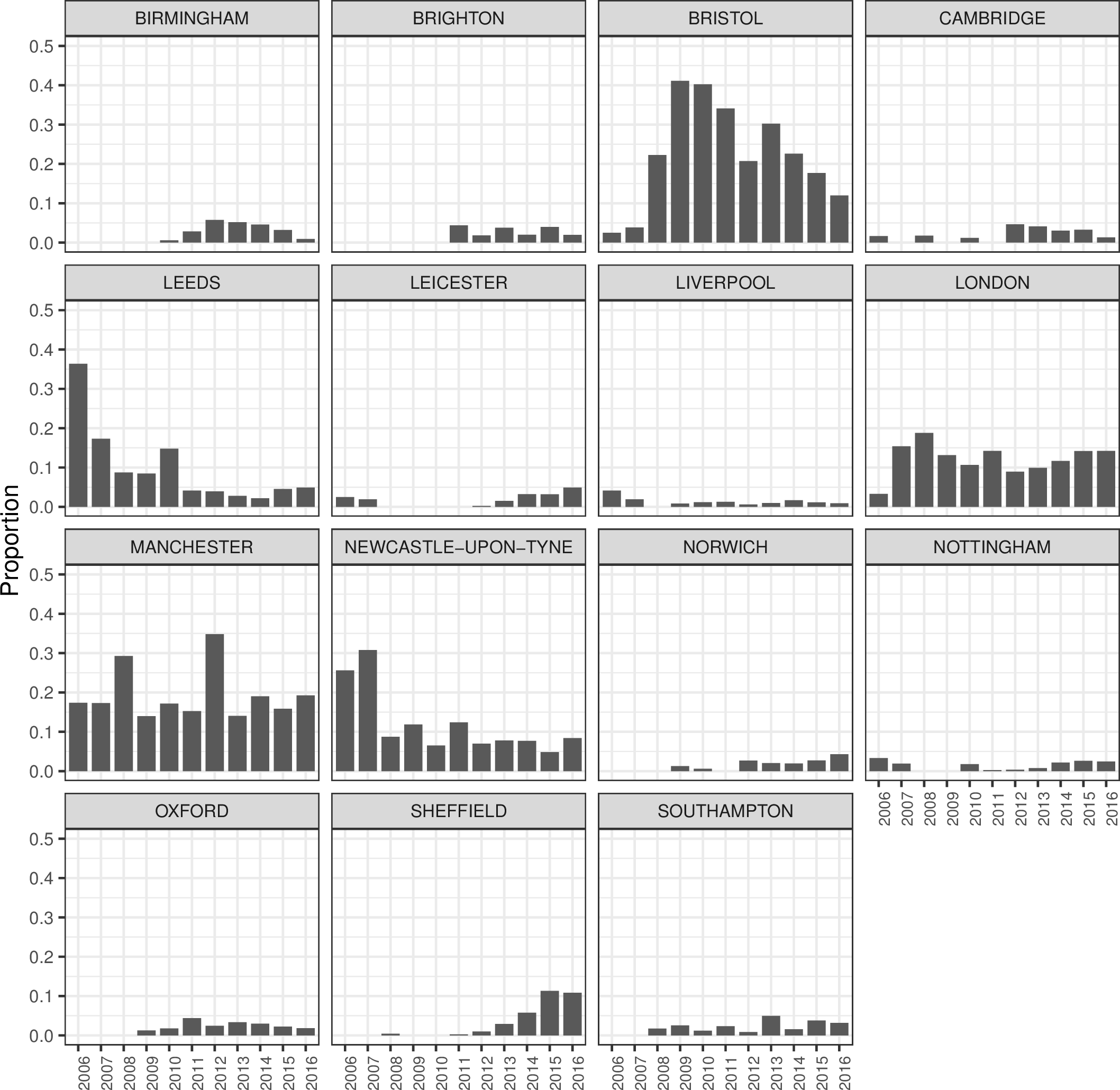

Supplement: S5 Fig — Only data for cities with over 90 detections during the 12-year period are shown. (TIF) [file ppat.1012703.s006.tif]

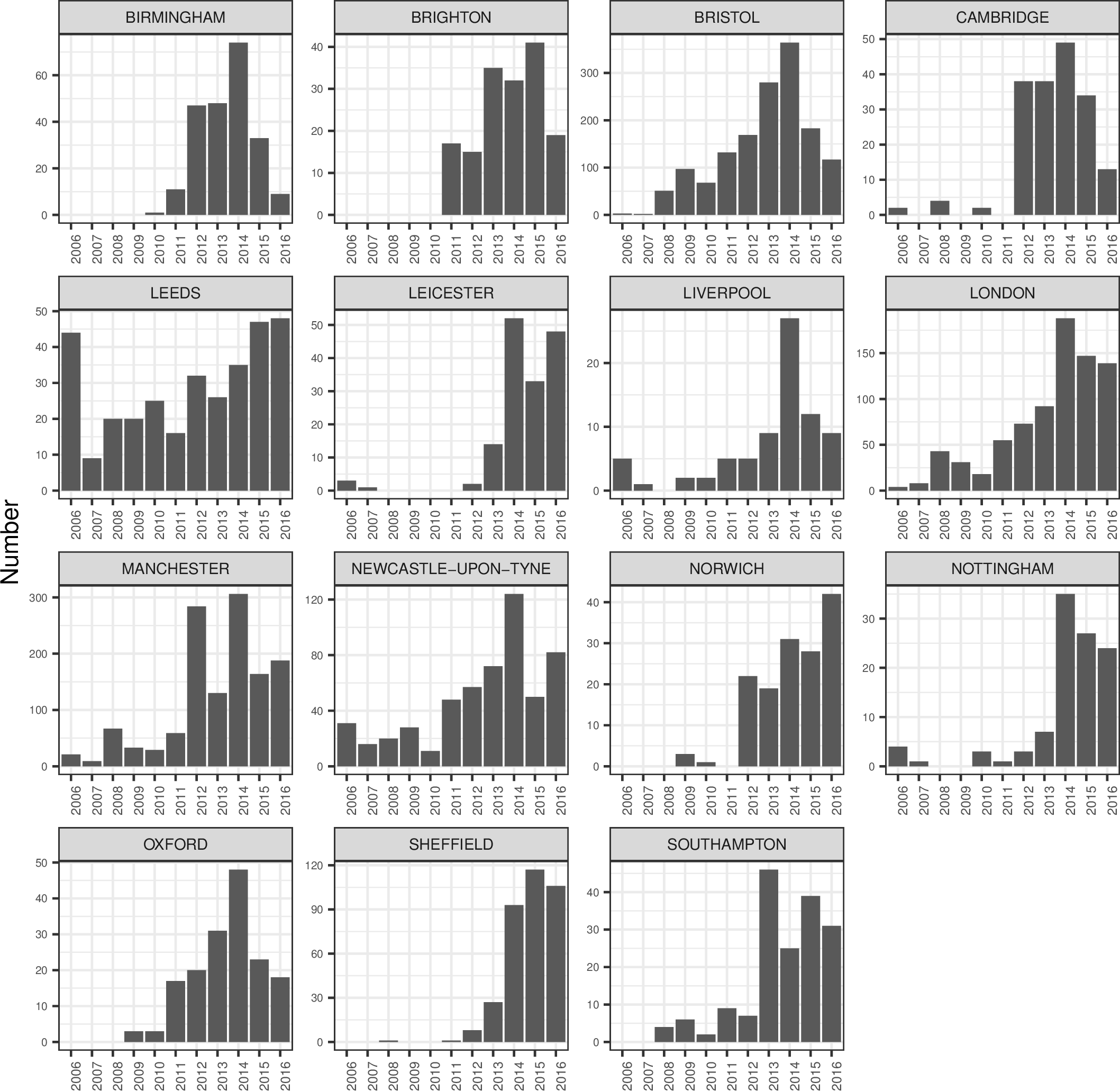

Supplement: S6 Fig — Only data for cities with over 90 detections during the 12-year period are shown. (TIF) [file ppat.1012703.s007.tif]

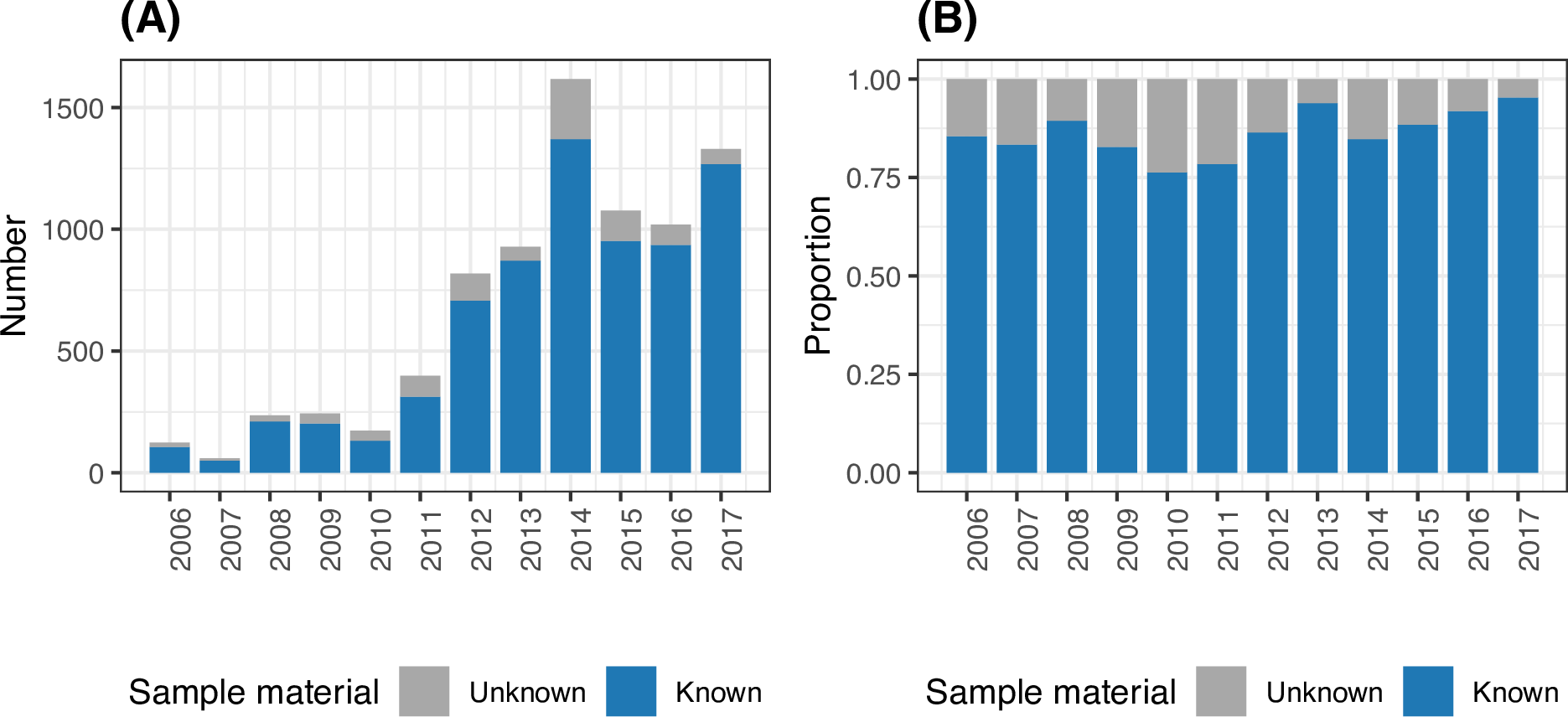

Supplement: S7 Fig — Total number (A) and proportion (B) of genotyped enterovirus-positive referrals from England, UK, submitted for genotyping to UKHSA, from 2006 to 2017, with and without information on sample sources. (TIF) [file ppat.1012703.s008.tif]

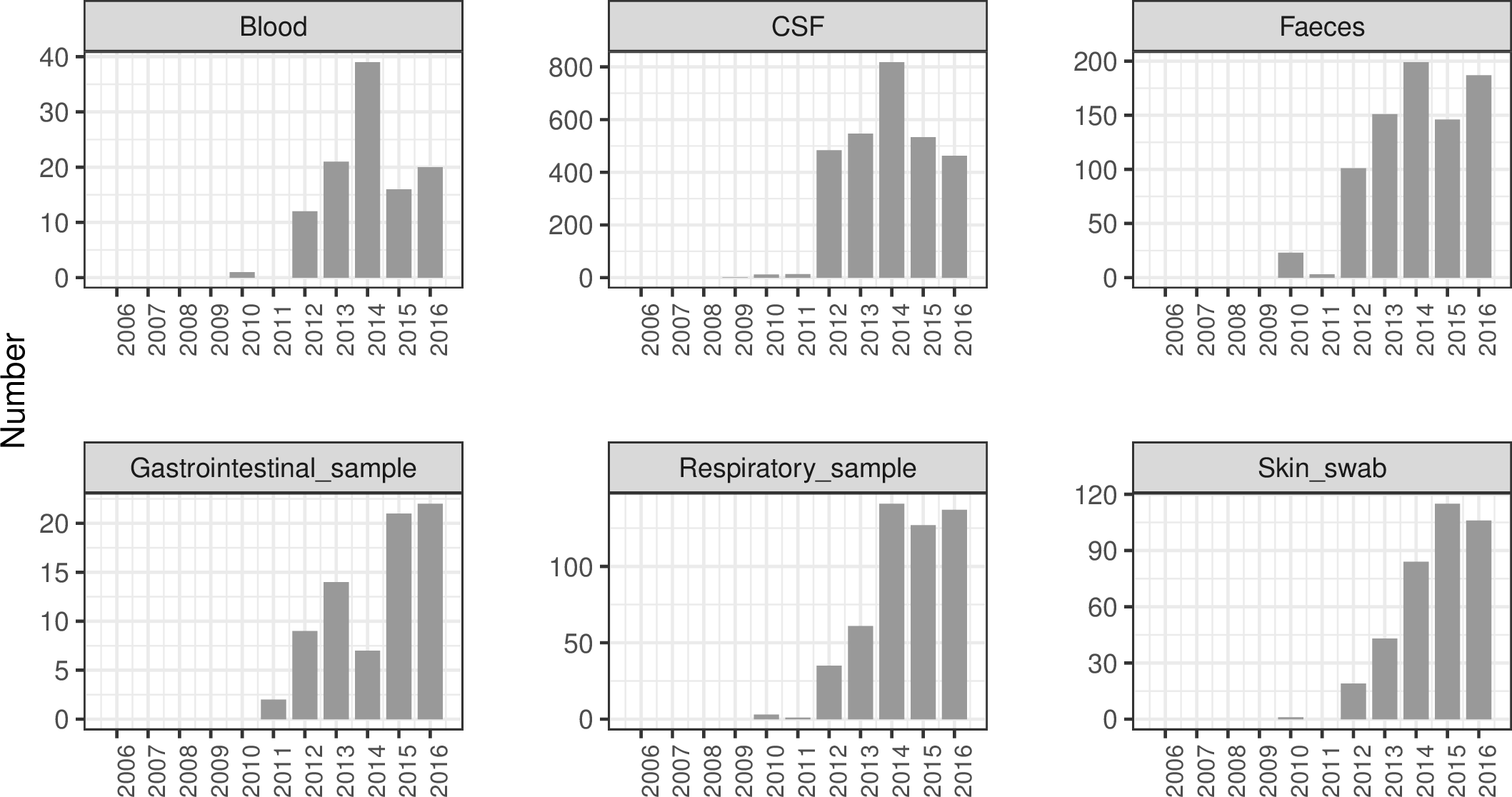

Supplement: S8 Fig — Sample sources recorded from genotyped enterovirus-positive referrals from England, UK, submitted for genotyping to UKHSA from 2006 to 2017. Only data for the 6 most frequently reported sample sources during 2006–2017 are shown. (TIF) [file ppat.1012703.s009.tif]

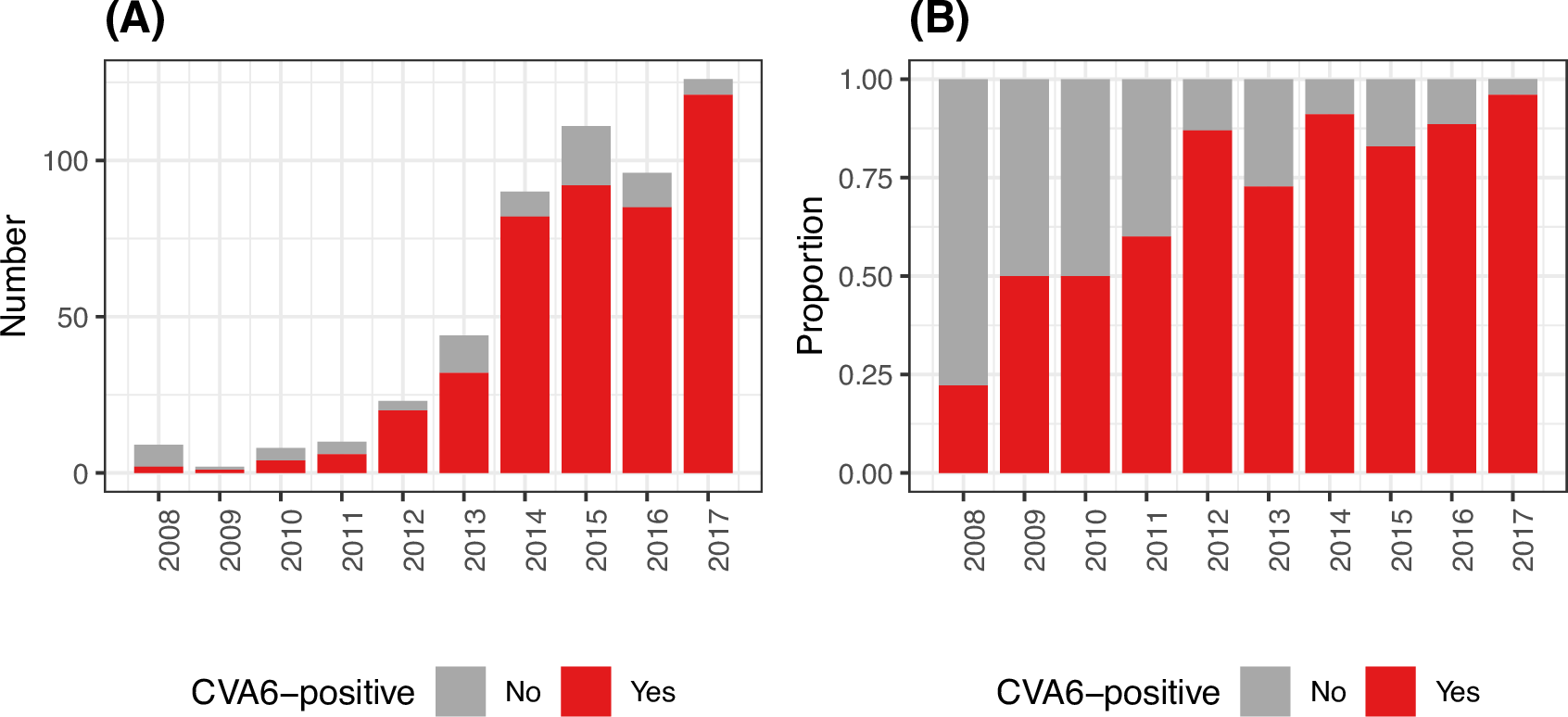

Supplement: S9 Fig — Total number (A) and proportion (B) of skin swabs positive for CVA6 each year among those referred from England, UK, submitted for genotyping to UKHSA from 2006 to 2017. (TIF) [file ppat.1012703.s010.tif]

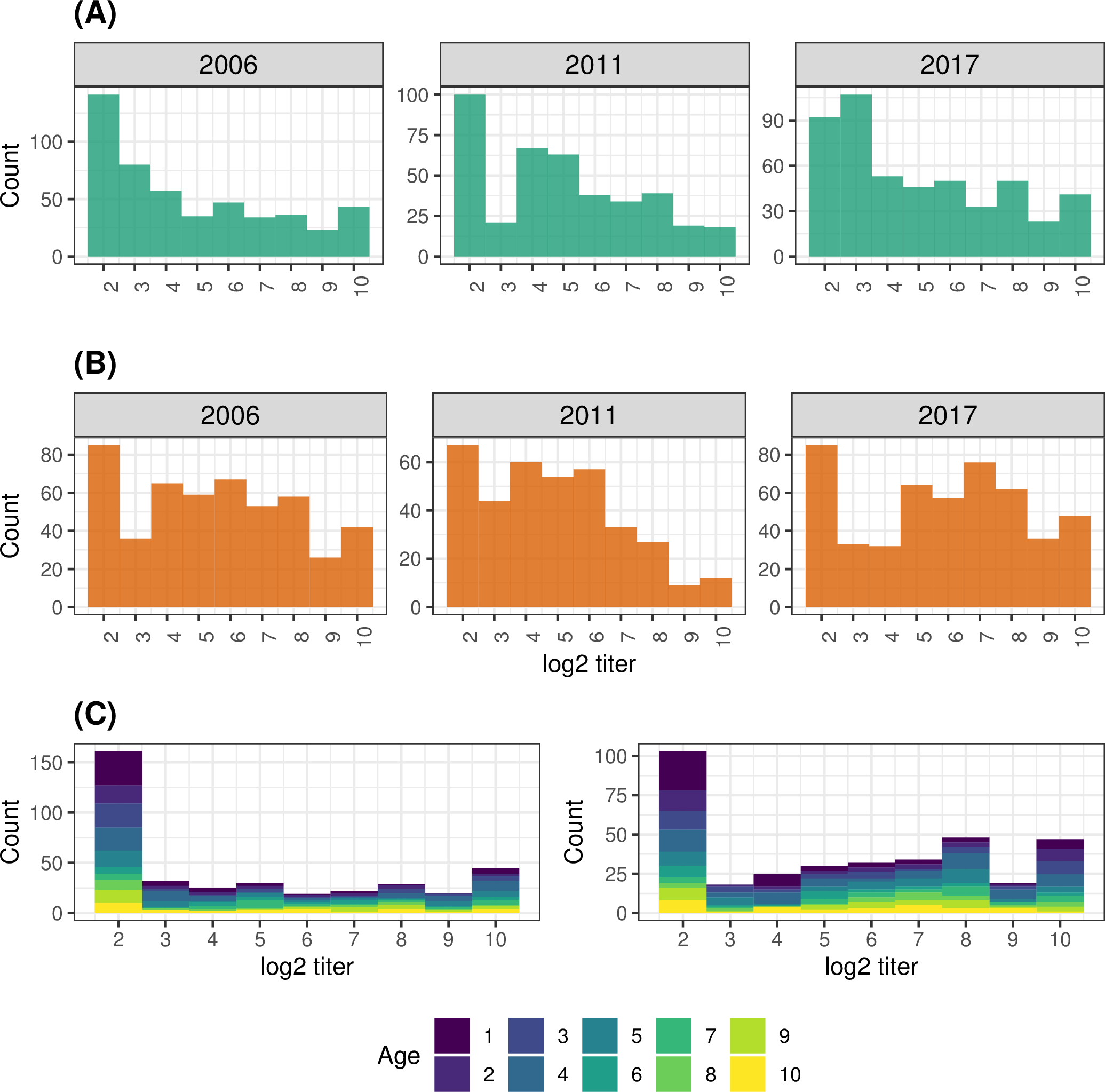

Supplement: S10 Fig — Antibody titer distributions for the three cross-sectional serosurveys (2006, 2011 and 2017) for EV-A71 (A) and CVA6 (B), and for the three serosurveys combined, but restricted to the younger population of 1–10 years old (C), for EV-A71 (C, left) and for CVA6 (C, right). (TIF) [file ppat.1012703.s011.tif]

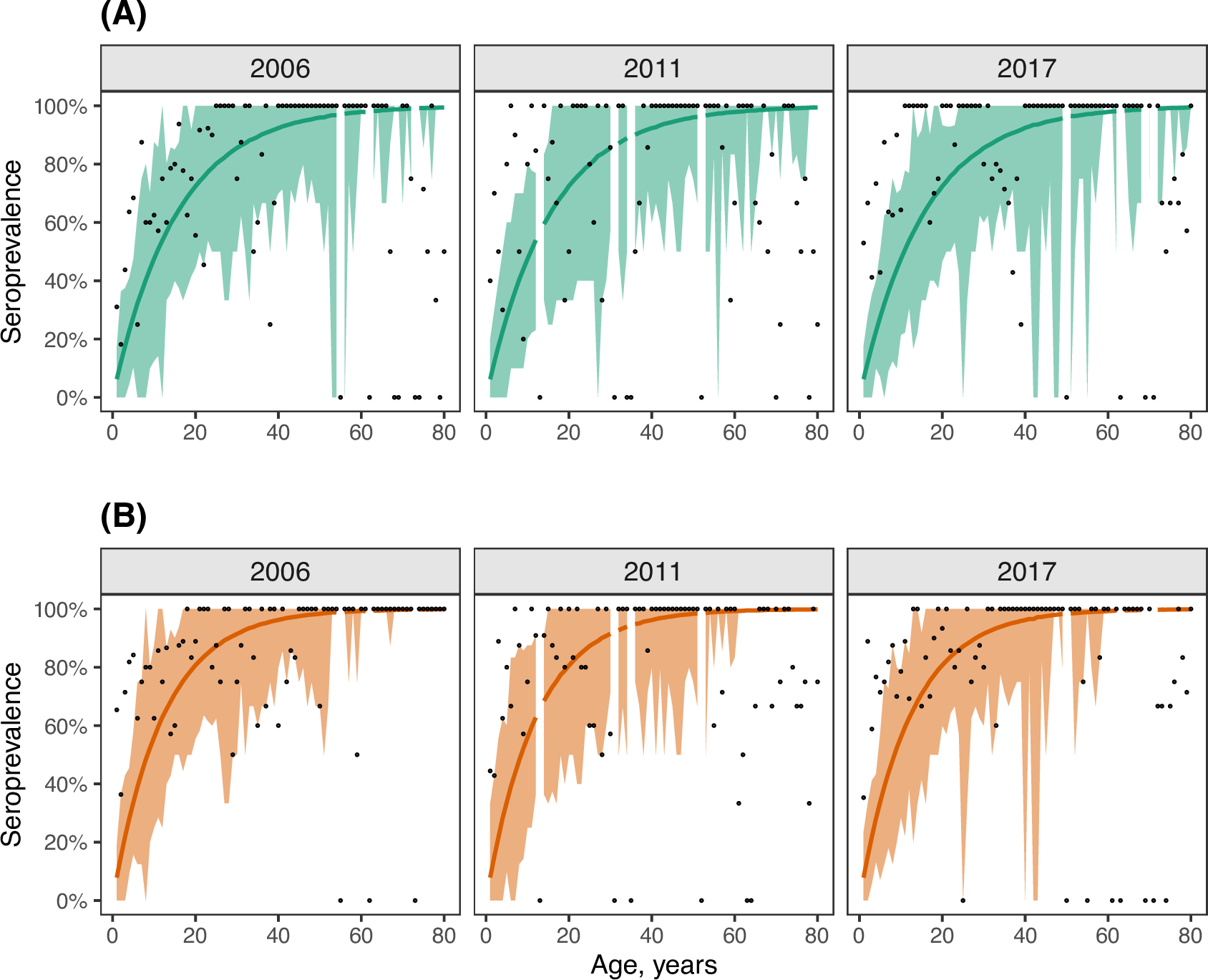

Supplement: S11 Fig — Model 1 (constant force of infection and no seroreversion) fit to data for EV-A71 (A) and CVA6 (B). The observed proportion of samples that were seropositive are shown as black circles. The solid lines and shaded area represent the model’s mean predicted seropositivity estimates and 95% Bayesian Credible Intervals. The gaps in the plots indicate absence of data in the corresponding age(s). (TIF) [file ppat.1012703.s012.tif]

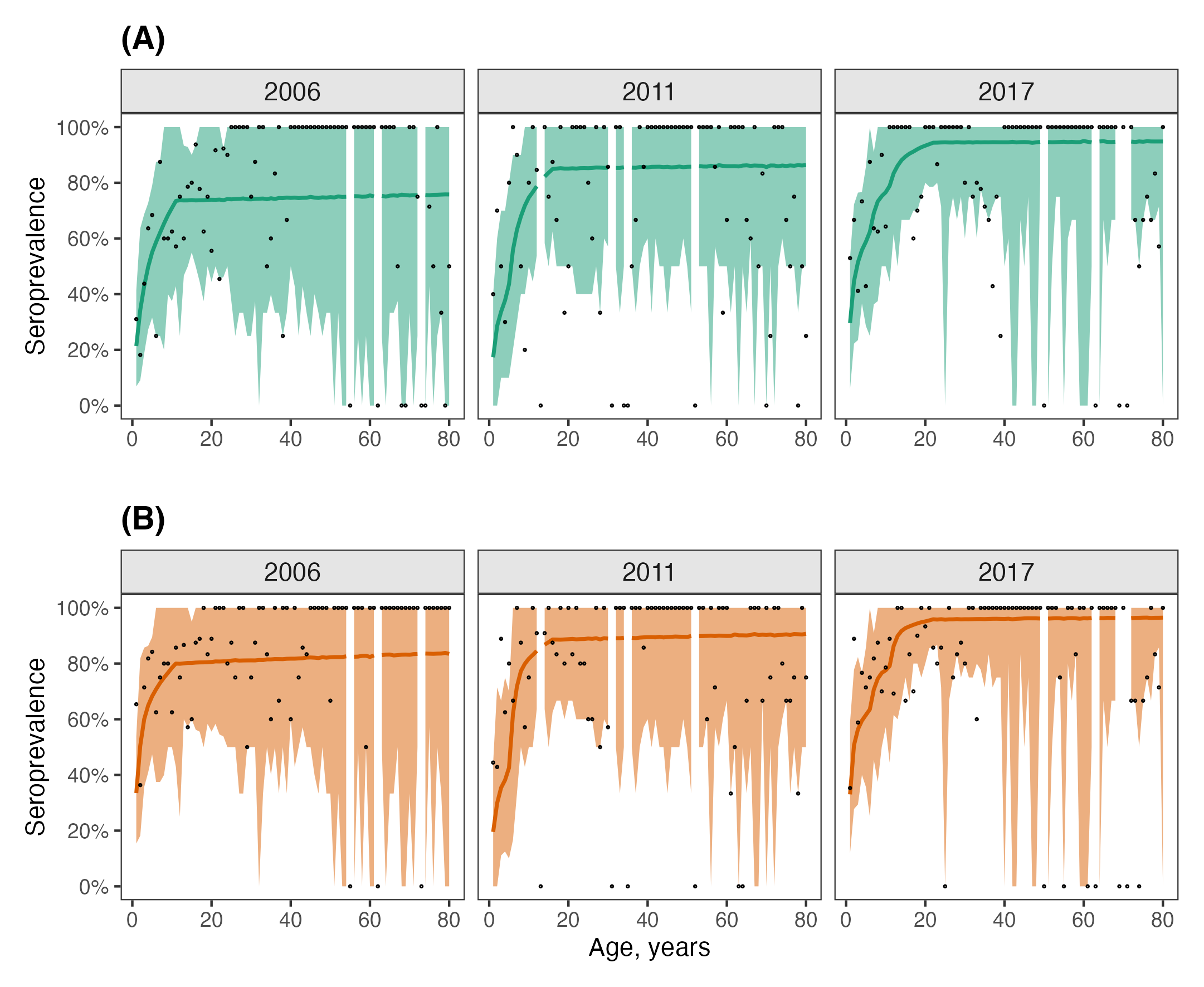

Supplement: S12 Fig — Model 3 (time-varying FOI model) fit to data for EV-A71 and CVA6. The observed proportion of samples that were seropositive are shown as black circles. The solid lines and shaded area represent the model’s mean predicted seropositivity estimates and 95% Bayesian Credible Intervals. The gaps in the plots indicate absence of data in the corresponding age(s). (TIF) [file ppat.1012703.s013.tif]

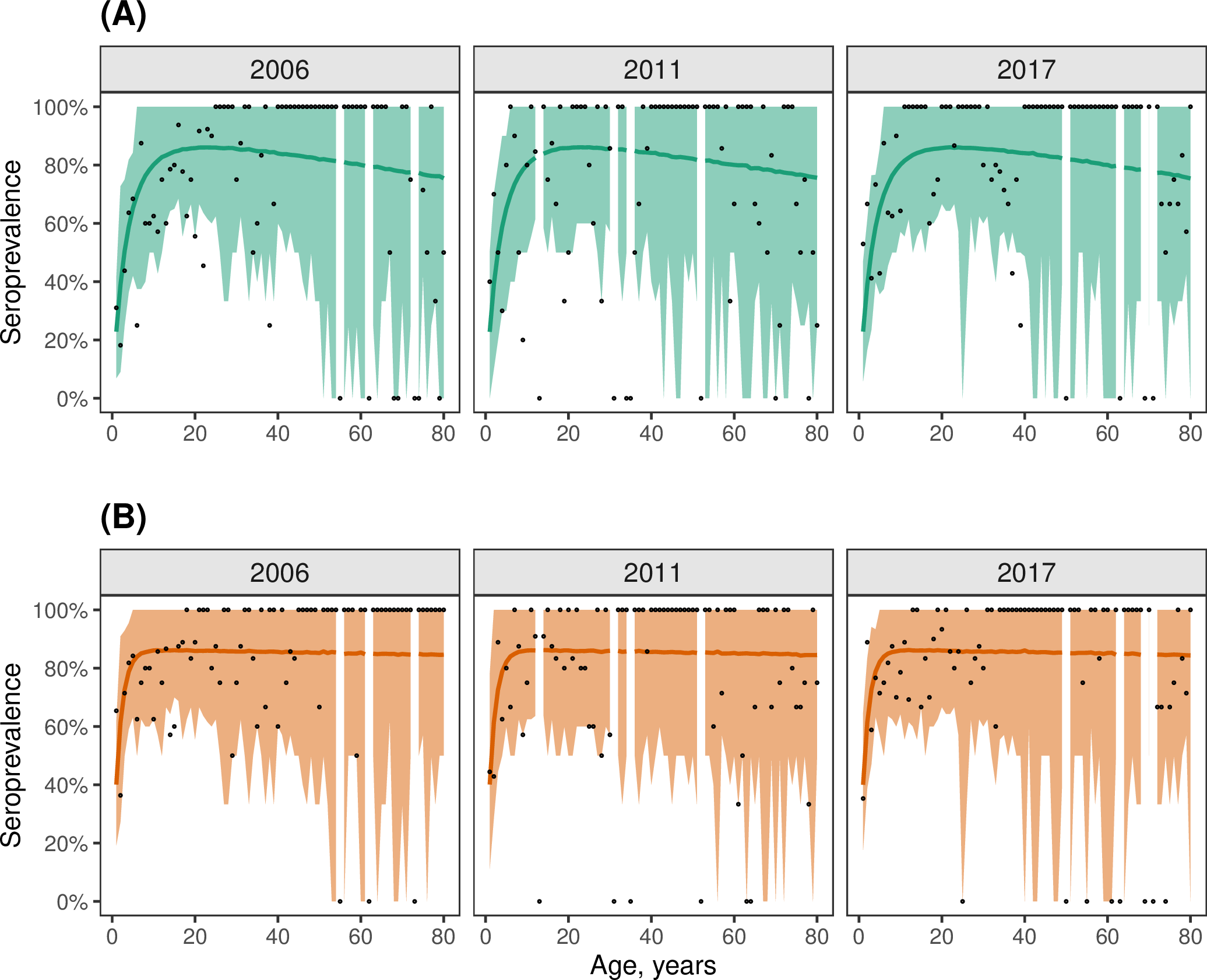

Supplement: S13 Fig — Model 6 (age-dependent force of infection and seroreversion) fit to data for EV-A71 (A) and CVA6 (B). The observed proportion of samples that were seropositive are shown as black circles. The solid lines and shaded area represent the model’s mean predicted seropositivity estimates and 95% Bayesian Credible Intervals. The gaps in the plots indicate absence of data in the corresponding age(s). (TIF) [file ppat.1012703.s014.tif]

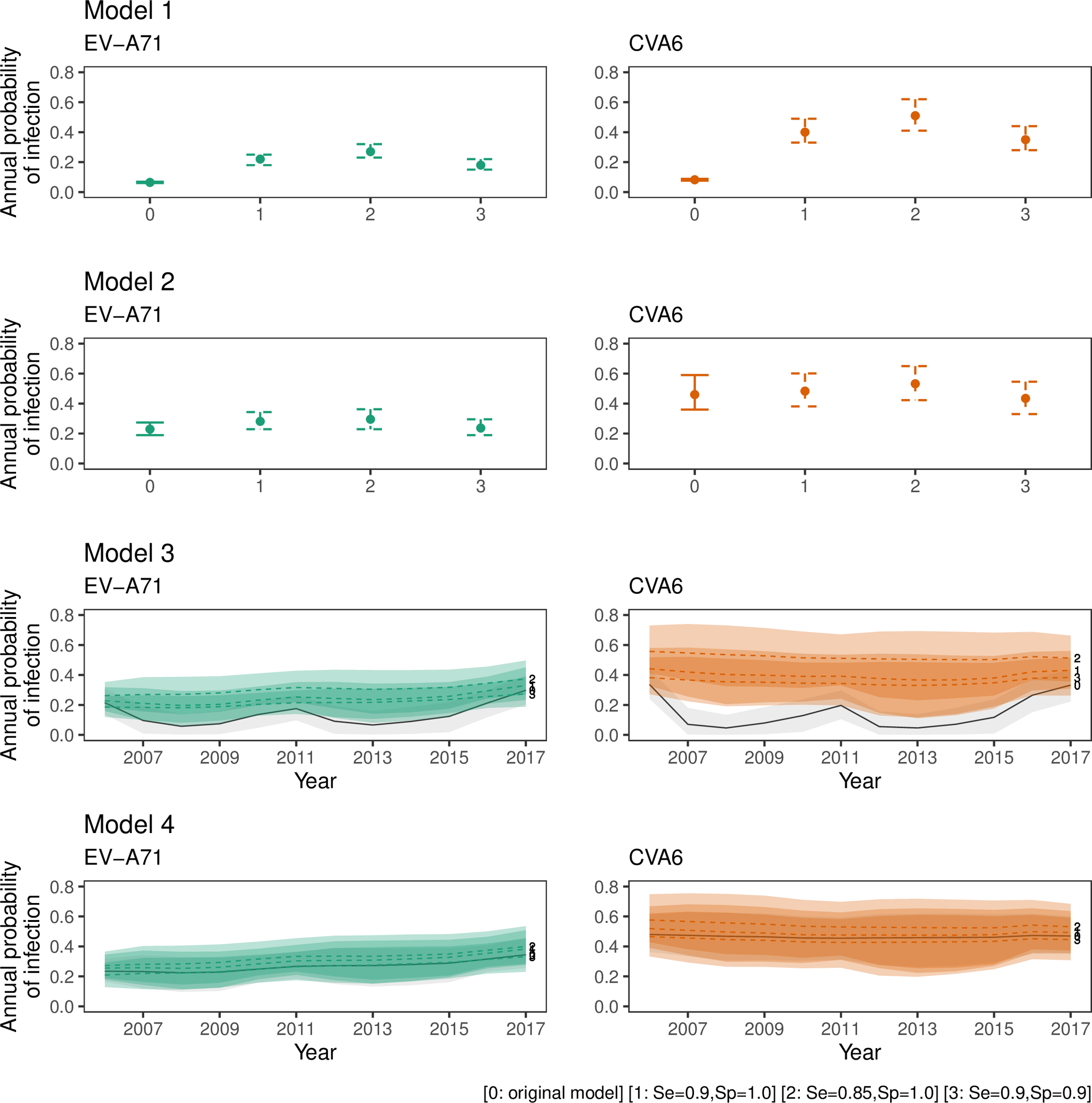

Supplement: S14 Fig — Estimates of the annual probability of infection for different values of sensitivity and specificity of the assay: 0, Se = 100%, Sp = 100% (results presented in the main text); 1, Se = 90%, Sp = 100%; 2, Se = 85%, Sp = 100%; and 3, Se = 90%, Sp = 90%. The corresponding parameter estimates for the seroreversion rate (ρ) for Models 2 and 4 are shown in S7 Table. (TIF) [file ppat.1012703.s015.tif]

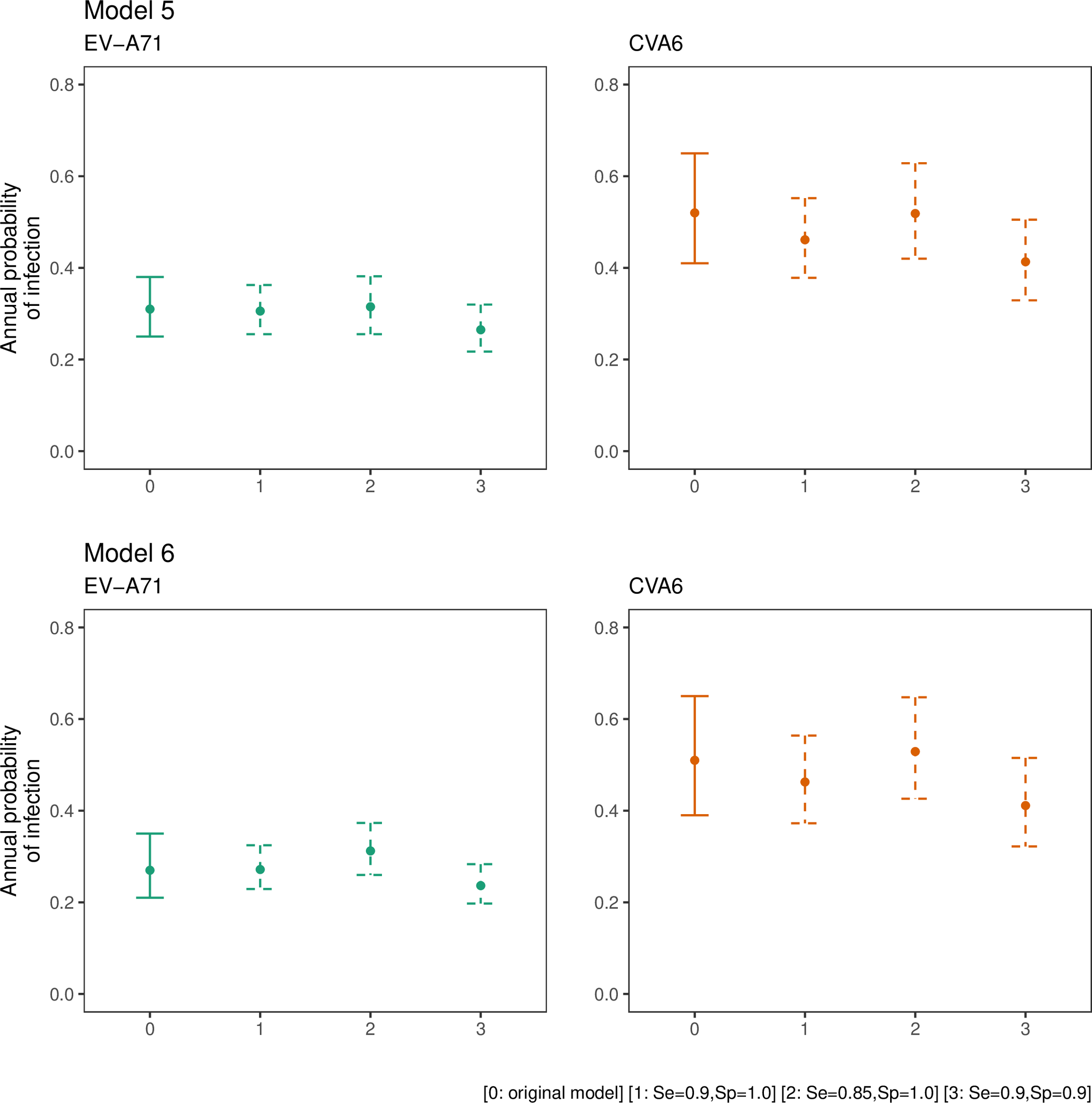

Supplement: S15 Fig — Estimates of the annual probability of infection at age 1 for different values of sensitivity and specificity of the assay: 0, Se = 100%, Sp = 100% (results presented in the main text); 1, Se = 90%, Sp = 100%; 2, Se = 85%, Sp = 100%; and 3, Se = 90%, Sp = 90%. The corresponding parameter estimates for β and ρ are listed in S8 Table. (TIF) [file ppat.1012703.s016.tif]
