# Supplementary material for: Enterovirus A71 and coxsackievirus A6 circulation in England, UK, 2006–2017: A mathematical modelling study using cross-sectional seroprevalence data
Source: PLoS Pathog. 2024 Nov 20;20(11):e1012703. doi: 10.1371/journal.ppat.1012703 (PMC11578500; doi:10.1371/journal.ppat.1012703)
Supplement: S1 Table — (DOCX) [file ppat.1012703.s017.docx]

| **Model** | **Prior** |
| --- | --- |
| 1 – Constant FOI (λ) | $\sim\mathrm{exponential} \left( 1 \right)$ |
| 2 – Constant FOI (λ) with seroreversion (ρ) | $\sim\mathrm{exponential} \left( 1 \right)$  $\sim\mathrm{exponential} \left( 20 \right)$ |
| 3 – Time-varying FOI (λ_t_) | $\lambda_{t>t_{1}}\vert\lambda_{t-1} \sim\mathrm{Normal}\left( \lambda_{t-1}, \sigma\right)$  $\lambda_{t=t_{1}}\sim\mathrm{exponential}\left( 1 \right)$  $\lambda_{c} \sim\mathrm{exponential} \left( 1 \right)$  $\sigma\sim\mathrm{exponential} \left( 1 \right)$ |
| 4 – Time-varying FOI (λ_t_) with seroreversion (ρ) | $\lambda_{t>t_{1}}\vert\lambda_{t-1} \sim\mathrm{Normal}\left( \lambda_{t-1}, \sigma\right)$  $\lambda_{t=t_{1}}\sim\mathrm{exponential}\left( 1 \right)$  $\lambda_{c} \sim\mathrm{exponential} \left( 1 \right)$  $\sigma\sim\mathrm{exponential} \left( 1 \right)$  $\sim\mathrm{exponential} \left( 20 \right)$ |
| 5 – Age-dependent constant FOI (λ_0_) | $\lambda_{1} \sim\mathrm{exponential} \left( 1 \right)$  $\sim\mathrm{exponential} \left( 20 \right)$ |
| 6 – Age-dependent constant FOI (λ_0_) with seroreversion (ρ) | $\lambda_{1} \sim\mathrm{exponential} \left( 1 \right)$  $\sim\mathrm{exponential} \left( 20 \right)$  $\sim\mathrm{exponential} \left( 20 \right)$ |
