# Supplementary material for: Enterovirus A71 and coxsackievirus A6 circulation in England, UK, 2006–2017: A mathematical modelling study using cross-sectional seroprevalence data
Source: PLoS Pathog. 2024 Nov 20;20(11):e1012703. doi: 10.1371/journal.ppat.1012703 (PMC11578500; doi:10.1371/journal.ppat.1012703)
Supplement: S2 Table — (DOCX) [file ppat.1012703.s018.docx]

|  | **EV-A71** | | | **CVA6** | | |
| --- | --- | --- | --- | --- | --- | --- |
| **Year**  **Age group** | **2006** | **2011** | **2017** | **2006** | **2011** | **2017** |
| 0-1 | 18 (55.6%) | 99 (66.7%) | 66 (48.5%) | 17 (70.6%) | 80 (62.5%) | 64 (60.9%) |
| 1-5 | 78 (41%) | 40 (47.5%) | 82 (61%) | 73 (67.1%) | 33 (60.6%) | 82 (67.1%) |
| 5-10 | 50 (62%) | 50 (68%) | 51 (66.7%) | 50 (78%) | 37 (78.4%) | 51 (76.5%) |
| 10-20 | 113 (74.3%) | 78 (83.3%) | 97 (85.6%) | 113 (77%) | 69 (89.9%) | 96 (82.3%) |
| 20-30 | 77 (83.1%) | 47 (83%) | 91 (97.8%) | 77 (90.9%) | 46 (82.6%) | 91 (86.8%) |
| 30-40 | 44 (72.7%) | 30 (76.7%) | 59 (71.2%) | 44 (86.4%) | 30 (86.7%) | 59 (94.9%) |
| 40-50 | 53 (100%) | 35 (100%) | 28 (100%) | 53 (86.8%) | 34 (100%) | 27 (100%) |
| 50-60 | 21 (100%) | 33 (84.8%) | 27 (100%) | 21 (85.7%) | 37 (78.4%) | 27 (88.9%) |
| 60-70 | 23 (73.9%) | 31 (77.4%) | 16 (100%) | 23 (100%) | 29 (69%) | 16 (93.8%) |
| 70-80 | 28 (64.3%) | 32 (59.4%) | 35 (74.3%) | 28 (100%) | 31 (74.2%) | 35 (74.3%) |
| 80-100 | 9 (33.3%) | 23 (56.5%) | 9 (88.9%) | 9 (88.9%) | 23 (73.9%) | 9 (88.9%) |

The age range in years, sample sizes and seroprevalence (in parenthesis) for each sampling time point.
