# Supplementary material for: Enterovirus A71 and coxsackievirus A6 circulation in England, UK, 2006–2017: A mathematical modelling study using cross-sectional seroprevalence data
Source: PLoS Pathog. 2024 Nov 20;20(11):e1012703. doi: 10.1371/journal.ppat.1012703 (PMC11578500; doi:10.1371/journal.ppat.1012703)
Supplement: S3 Table — (DOCX) [file ppat.1012703.s019.docx]

| **Model** | **EV-A71: mean (95% Credible Interval)** | **CVA6: mean (95% Credible Interval)** |
| --- | --- | --- |
| 1 – Constant FOI (λ) | λ = 0.064 (0.06 – 0.07) | λ = 0.082 (0.076 – 0.088) |
| 2 – Constant FOI (λ) with seroreversion (ρ) | λ = 0.3 (0.24 – 0.37)  ρ = 0.062 (0.04 – 0.086) | λ = 0.58 (0.43 – 0.78)  ρ = 0.1 (0.067 – 0.14) |
| 5 – Age-dependent constant FOI (λ_1_) | $\lambda_{1}$ = 0.31 (0.25 – 0.38)  β = 0.19 (0.15 – 0.25) | $\lambda_{1}$ = 0.52 (0.41 – 0.65)  β = 0.3 (0.22 – 0.41) |
| 6 – Age-dependent constant FOI (λ_1_) with seroreversion (ρ) | $\lambda_{1}$ = 0.27 (0.21 – 0.35)  β = 0.08 (0.003 – 0.17)  ρ = 0.015 (0.0009 – 0.06) | $\lambda_{1}$ = 0.51 (0.39 – 0.65)  β = 0.001 (0.00004 – 0.005)  ρ = 0.12 (0.072 – 0.19) |

Parameter estimates resulting from the different catalytic models with constant FOI over time using priors defined in S1 Table.
