# Supplementary material for: Enterovirus A71 and coxsackievirus A6 circulation in England, UK, 2006–2017: A mathematical modelling study using cross-sectional seroprevalence data
Source: PLoS Pathog. 2024 Nov 20;20(11):e1012703. doi: 10.1371/journal.ppat.1012703 (PMC11578500; doi:10.1371/journal.ppat.1012703)
Supplement: S4 Table — (DOCX) [file ppat.1012703.s020.docx]

| **Model** | **EV-A71: mean (95% Credible Interval)** | **CVA6: mean (95% Credible Interval)** |
| --- | --- | --- |
| 1 – Time-constant FOI (λ) | λ = 0.064 (0.06 – 0.07) | λ = 0.082 (0.076 – 0.088) |
| 2 – Time-constant FOI (λ) with seroreversion (ρ) | λ = 0.28 (0.23 – 0.35)  ρ = 0.06 (0.04 – 0.08) | λ = 0.52 (0.4 – 0.67)  ρ = 0.086 (0.06 – 0.12) |
| 5 – Age-dependent time-constant FOI (λ_1_) | $\lambda_{1}$ = 0.3 (0.25 – 0.37)  β = 0.18 (0.14 – 0.24) | $\lambda_{1}$ = 0.49 (0.38 – 0.61)  β = 0.28 (0.2 – 0.37) |
| 6 – Age-dependent time-constant FOI (λ_1_) with seroreversion (ρ) | $\lambda_{1}$ = 0.26 (0.2 – 0.33)  β = 0.075 (0.004 – 0.16)  ρ = 0.015 (0.0012 – 0.06) | $\lambda_{1}$ = 0.47 (0.37 – 0.6)  β = 0.0017 (0.000057 – 0.0054)  ρ = 0.11 (0.065 – 0.16) |

These are parameter estimates from sensitivity analysis on the prior for λ for the time-constant FOI models. See Supporting Information for detailed description of sensitivity analyses.
