# Supplementary material for: Enterovirus A71 and coxsackievirus A6 circulation in England, UK, 2006–2017: A mathematical modelling study using cross-sectional seroprevalence data
Source: PLoS Pathog. 2024 Nov 20;20(11):e1012703. doi: 10.1371/journal.ppat.1012703 (PMC11578500; doi:10.1371/journal.ppat.1012703)
Supplement: S5 Table — (DOCX) [file ppat.1012703.s021.docx]

| **Model** | $\boldsymbol{\sim exponential(1)}$  **mean (95% Credible Interval)** | $\boldsymbol{\sim exponential(20)}$  **mean (95% Credible Interval)** |
| --- | --- | --- |
| 2 – Time-constant FOI (λ) with seroreversion (ρ) | λ = 0.26 (0.21 – 0.32)  ρ = 0.06 (0.04 – 0.09) | λ = 0.26 (0.21 – 0.32)  ρ = 0.062 (0.04 – 0.086) |
| 4 – Time-varying FOI (λ_t_) with seroreversion (ρ) | ρ = 0.06 (0.04 – 0.08)  σ = 0.06 (0.01 – 0.18)  $\lambda_{c}$= 0.6 (0.05 – 0.97) | ρ = 0.06 (0.04 – 0.08)  σ = 0.06 (0.016 – 0.16)  $\lambda_{c}$= 0.9 (0.05 - 3.5) |
| 6 – Age-dependent time-constant FOI (λ_1_) with seroreversion (ρ) | $\lambda_{1}$ = 0.23 (0.18 – 0.29)  β = 0.06 (0.001 – 0.17)  ρ = 0.023 (0.001 – 0.04) | $\lambda_{1}$ = 0.23 (0.19 – 0.29)  β = 0.06 (0.001 – 0.17)  ρ = 0.015 (0.0009 – 0.06) |

These are parameter estimates from sensitivity analysis on the prior for ρ for the models assuming seroreversion. See Supporting Information for detailed description of sensitivity analyses.
