# Supplementary material for: Enterovirus A71 and coxsackievirus A6 circulation in England, UK, 2006–2017: A mathematical modelling study using cross-sectional seroprevalence data
Source: PLoS Pathog. 2024 Nov 20;20(11):e1012703. doi: 10.1371/journal.ppat.1012703 (PMC11578500; doi:10.1371/journal.ppat.1012703)
Supplement: S6 Table — (DOCX) [file ppat.1012703.s022.docx]

| **Model** | $\boldsymbol{\sim exponential(1)}$  **mean (95% Credible Interval)** | $\boldsymbol{\sim exponential(20)}$  **mean (95% Credible Interval)** |
| --- | --- | --- |
| 2 – Time-constant FOI (λ) with seroreversion (ρ) | λ = 0.46 (0.36 – 0.58)  ρ = 0.11 (0.07 – 0.16) | λ = 0.46 (0.36 – 0.58)  ρ = 0.1 (0.067 – 0.14) |
| 4 – Time-varying FOI (λ_t_) with seroreversion (ρ) | ρ = 0.11 (0.07 – 0.17)  σ = 0.07 (0.003 – 0.3)  $\lambda_{c}$= 0.63 (0.02 – 0.97) | ρ = 0.1 (0.06 – 0.14)  σ = 0.07 (0.003 – 0.3)  $\lambda_{c}$= 0.98 (0.026 – 3.6) |
| 6 – Age-dependent time-constant FOI (λ_1_) with seroreversion (ρ) | $\lambda_{1}$ = 0.42 (0.34 – 0.52)  β = 0.001 (0.00003 – 0.004)  ρ = 0.15 (0.08 – 0.26) | $\lambda_{1}$ = 0.42 (0.34 – 0.52)  β = 0.001 (0.00004 – 0.004)  ρ = 0.12 (0.072 – 0.19) |

These are parameter estimates from sensitivity analysis on the prior for ρ for the models assuming seroreversion. See Supporting Information for detailed description of sensitivity analyses.
