# Supplementary material for: Enterovirus A71 and coxsackievirus A6 circulation in England, UK, 2006–2017: A mathematical modelling study using cross-sectional seroprevalence data
Source: PLoS Pathog. 2024 Nov 20;20(11):e1012703. doi: 10.1371/journal.ppat.1012703 (PMC11578500; doi:10.1371/journal.ppat.1012703)
Supplement: S7 Table — Sensitivity (Se) and specificity (Sp) values were labelled as: 0, Se = 100%, Sp = 100% (results presented in the main text); 1, Se = 90%, Sp = 100%; 2, Se = 85%, Sp = 100%; and 3, Se = 90%, Sp = 90%. (DOCX) [file ppat.1012703.s023.docx]

| **Model** | **EV-A71**  **mean (95% Credible Interval)** | **CVA6**  **mean (95% Credible Interval)** |
| --- | --- | --- |
| 2 – Time-constant FOI (λ) with seroreversion (ρ) | 0: 0.062 (0.04 – 0.086)  1: 0.03 (0.017 – 0.047)  2: 0.011 (0.0014 – 0.024)  3: 0.026 (0.014 - 0.042) | 0: 0.1 (0.067 – 0.14)  1: 0.036 (0.016 – 0.064)  2: 0.007 (0.0002 – 0.022)  3: 0.033 (0.014 – 0.06) |
| 4 – Time-varying FOI (λ_t_) with seroreversion (ρ) | 0: 0.06 (0.04 – 0.08)  1: 0.027 (0.014 – 0.044)  2: 0.01 (0.001 – 0.024)  3: 0.025 (0.012 – 0.041) | 0: 0.1 (0.06 – 0.14)  1: 0.036 (0.015 – 0.065)  2: 0.007 (0.0002 – 0.02)  3: 0.033 (0.013 – 0.063) |

These are parameter estimates from sensitivity analysis on the prior for λ for the time-constant FOI models. See Supporting Information for detailed description of sensitivity analyses.
