# Supplementary material for: Enterovirus A71 and coxsackievirus A6 circulation in England, UK, 2006–2017: A mathematical modelling study using cross-sectional seroprevalence data
Source: PLoS Pathog. 2024 Nov 20;20(11):e1012703. doi: 10.1371/journal.ppat.1012703 (PMC11578500; doi:10.1371/journal.ppat.1012703)
Supplement: S8 Table — Sensitivity (Se) and specificity (Sp) values were labelled as: 0, Se = 100%, Sp = 100% (results presented in the main text); 1, Se = 90%, Sp = 100%; 2, Se = 85%, Sp = 100%; and 3, Se = 90%, Sp = 90%. (DOCX) [file ppat.1012703.s024.docx]

| **ρ** |  |  |
| --- | --- | --- |
| **Model 6** | **EV-A71**  **mean (95% Credible Interval)** | **CVA6**  **mean (95% Credible Interval)** |
| original model | 0.015 (0.0009 - 0.06) | 0.12 (0.07 - 0.19) |
| Se = 0.9, Sp = 1.0 | 0.006 (0.001 - 0.02) | 0.03 (0.0002 - 0.08) |
| Se = 0.85, Sp = 1.0 | 0.004 (0.0005 - 0.01) | 0.0048 (0.00006 - 0.02) |
| Se = 0.9, Sp = 0.9 | 0.007 (0.002 - 0.02) | 0.02 (0.0002 - 0.07) |
| **β** |  |  |
| **Model 5** | **EV-A71**  **mean (95% Credible Interval)** | **CVA6**  **mean (95% Credible Interval)** |
| original model | 0.19 (0.15 - 0.25) | 0.3 (0.22 - 0.41) |
| Se = 0.9, Sp = 1.0 | 0.15 (0.1 - 0.2) | 0.2 (0.12 - 0.3) |
| Se = 0.85, Sp = 1.0 | 0.08 (0.01 - 0.15) | 0.06 (0.002 - 0.17) |
| Se = 0.9, Sp = 0.9 | 0.12 (0.08 - 0.18) | 0.17 (0.1 - 0.27) |
| **Model 6** | **EV-A71**  **mean (95% Credible Interval)** | **CVA6**  **mean (95% Credible Interval)** |
| original model | 0.08 (0.003 - 0.17) | 0.0015 (0.00004 - 0.005) |
| Se = 0.9, Sp = 1.0 | 0.05 (0.01 - 0.1) | 0.02 (0.0001 - 0.2) |
| Se = 0.85, Sp = 1.0 | 0.04 (0.006 - 0.1) | 0.02 (0.0004 - 0.1) |
| Se = 0.9, Sp = 0.9 | 0.04 (0.01 - 0.09) | 0.027 (0.0002 - 0.18) |

The original model had sensitivity (Se) = 100% and specificity (Sp) = 100%, and the results are presented in the main text.
