# Supplementary material for: Enterovirus A71 and coxsackievirus A6 circulation in England, UK, 2006–2017: A mathematical modelling study using cross-sectional seroprevalence data
Source: PLoS Pathog. 2024 Nov 20;20(11):e1012703. doi: 10.1371/journal.ppat.1012703 (PMC11578500; doi:10.1371/journal.ppat.1012703)
Supplement: S9 Table — Models are compared and ranked across assay accuracy parameters: sensitivity (Se) and specificity (Sp). (DOCX) [file ppat.1012703.s025.docx]

| **EV-A71** | | | | | | | | | | | | |
| --- | --- | --- | --- | --- | --- | --- | --- | --- | --- | --- | --- | --- |
|  | **Se=100%, Sp=100%** | | | **Se=90%, Sp=100%** | | | **Se=85%, Sp=100%** | | | **Se=90%, Sp=90%** | | |
| **Model** | **elpd_diff** | **se_diff** | **rank** | **elpd_diff** | **se_diff** | **rank** | **elpd_diff** | **se_diff** | **rank** | **elpd_diff** | **se_diff** | **rank** |
| 1 | 260.4 | 40.7 | 6 | 24.6 | 9.7 | 6 | 4.2 | 1.5 | 6 | 21.1 | 7.8 | 6 |
| 2 | 3.9 | 2.54 | 4 | 5.2 | 1.9 | 4 | 3.7 | 1.1 | 5 | 5.4 | 2.1 | 4 |
| 3 | 23.6 | 12.6 | 5 | 9.3 | 2.3 | 5 | 1 | 0.4 | 2 | 8.2 | 2.7 | 5 |
| 4 | 0 | 0 | 1 | 1.8 | 0.4 | 2 | 1.2 | 0.2 | 3 | 2.8 | 0.6 | 2 |
| 5 | 1.2 | 0.1 | 2 | 2.2 | 0.9 | 3 | 1.8 | 0.06 | 4 | 4 | 1.7 | 3 |
| 6 | 1.6 | 0.3 | 3 | 0 | 0 | 1 | 0 | 0 | 1 | 0 | 0 | 1 |
| **CVA6** | | | | | | | | | | | | |
|  | **Se=100%, Sp=100%** | | | **Se=90%, Sp=100%** | | | **Se=85%, Sp=100%** | | | **Se=90%, Sp=90%** | | |
| **Model** | **elpd_diff** | **se_diff** | **rank** | **elpd_diff** | **se_diff** | **rank** | **elpd_diff** | **se_diff** | **rank** | **elpd_diff** | **se_diff** | **rank** |
| 1 | 261.1 | 48.3 | 6 | 10.9 | 4.76 | 6 | 5.7 | 2.3 | 6 | 9.4 | 4.5 | 6 |
| 2 | 2.4 | 0.15 | 2 | 2 | 1.1 | 2 | 1.6 | 0.6 | 3 | 1.7 | 1.2 | 2 |
| 3 | 44.7 | 13.67 | 5 | 10.9 | 4.28 | 5 | 2.5 | 1.2 | 4 | 9.2 | 4.3 | 5 |
| 4 | 3.4 | 2.6 | 4 | 3.2 | 1.6 | 4 | 3.1 | 1.4 | 5 | 3.2 | 2.3 | 4 |
| 5 | 0 | 0 | 1 | 0 | 0 | 1 | 0 | 0 | 1 | 0 | 0 | 1 |
| 6 | 3.1 | 0.27 | 3 | 3.1 | 1.4 | 3 | 1.1 | 0.4 | 2 | 2.6 | 1.7 | 3 |

Model comparison and ranking of the fitted catalytic models using the approximate leave-one-out cross-validation (LOO-CV) method. LOO-CV calculates the expected log pointwise predictive density (ELPD) for each model, which is a measure of the overall model fit accounting for model complexity. Model ranking is based on the differences in the ELPD and standard error estimates (‘*elpd_diff*’, and ‘*se_diff*’, respectively), where the differences are calculated relative to the model with the largest ELPD.
